# Generation of heterozygous *PKD1* mutant pigs exhibiting early-onset renal cyst formation

**DOI:** 10.1038/s41374-021-00717-z

**Published:** 2022-01-03

**Authors:** Masahito Watanabe, Kazuhiro Umeyama, Kazuaki Nakano, Hitomi Matsunari, Toru Fukuda, Kei Matsumoto, Susumu Tajiri, Shuichiro Yamanaka, Koki Hasegawa, Kazutoshi Okamoto, Ayuko Uchikura, Shuko Takayanagi, Masaki Nagaya, Takashi Yokoo, Hiromitsu Nakauchi, Hiroshi Nagashima

**Affiliations:** 1 Meiji University International Institute for Bio-Resource Research, 1-1-1 Higashimita, Tama-ku, Kawasaki, Kanagawa 214-8571 Japan; 2 Laboratory of Medical Bioengineering, Department of Life Sciences, School of Agriculture, Meiji University, 1-1-1 Higashimita, Tama-ku, Kawasaki, Kanagawa 214-8571 Japan; 3 Division of Nephrology and Hypertension, Department of Internal Medicine, The Jikei University School of Medicine, 3-25-8, Nishi-Shimbashi, Minato-ku, Tokyo, 105-8461 Japan; 4 Division of Stem Cell Therapy, Distinguished Professor Unit, Institute of Medical Science, University of Tokyo, 4-6-1 Shirokanedai, Minato-ku, Tokyo, 108-8639 Japan; 5 Institute for Stem Cell Biology and Regenerative Medicine, Department of Genetics, Stanford University School of Medicine, 265 Campus Drive, Stanford, CA 94305 USA

**Keywords:** Experimental models of disease, End-stage renal disease

## Abstract

Autosomal dominant polycystic kidney disease (ADPKD) is the most common inherited kidney disease, manifesting as the progressive development of fluid-filled renal cysts. In approximately half of all patients with ADPKD, end-stage renal disease results in decreased renal function. In this study, we used CRISPR-Cas9 and somatic cell cloning to produce pigs with the unique mutation c.152_153insG *(PKD1*^*insG/+*^). Pathological analysis of founder cloned animals and progeny revealed that *PKD1*^*insG/+*^ pigs developed many pathological conditions similar to those of patients with heterozygous mutations in *PKD1*. Pathological similarities included the formation of macroscopic renal cysts at the neonatal stage, number and cystogenic dynamics of the renal cysts formed, interstitial fibrosis of the renal tissue, and presence of a premature asymptomatic stage. Our findings demonstrate that *PKD1*^*insG/+*^ pigs recapitulate the characteristic symptoms of ADPKD.

## Abstract (web summary)

Autosomal dominant polycystic kidney disease (ADPKD) is a genetic disorder characterized by the formation of cysts within the kidneys. The authors generated *PKD1* heterozygous knockout (*PKD1*^*insG/+*^) pigs by CRISPR-Cas9 and somatic cell cloning techniques. The founder cloned animals and progeny showed the renal cyst formation from the neonatal stage, interstitial fibrosis of the renal tissue, and the presence of a premature asymptomatic stage. Their findings demonstrate that *PKD1*^*insG/+*^ pigs recapitulate the characteristic symptoms of ADPKD.

## Introduction

Autosomal dominant polycystic kidney disease (ADPKD) affects one in every 500–1000 people worldwide and is the most common inherited kidney disease^1–3^. The characteristic pathology of ADPKD is progressive development of fluid-filled cysts that generally arise bilaterally in the kidney tissue. Decreased renal function accompanying renal cyst formation occurs in approximately half of patients succumbing to end-stage renal disease at approximately age 60 years^1,4,5^. Extrarenal manifestations in patients with ADPKD include hypertension, hepatic and pancreatic cysts, valvular heart disease, and intracranial aneurysm^6–8^. Although the relatively new drug tolvaptan is available and suppresses the progression of renal cyst formation, a curative therapy for this disease has not been developed^9^.

The polycystic kidney disease (PKD) genes *PKD1* and *PKD2* code for polycystin 1 (PC1) and polycystin 2 (PC2), respectively, and are known as the causal genes of ADPKD. Mutations in *PKD1* and *PKD2* are responsible for approximately 85% and 15%, respectively, of all ADPKD cases^10^. Although the clinical pathologies accompanying these mutations are similar, *PKD1*-associated ADPKD exhibits an earlier onset and a more severe disease course than *PKD2*-associated ADPKD^4^.

The pathologies and etiologies of ADPKD have been investigated using PKD animal models, such as rats harboring spontaneous mutations in the PKD genes *Pkhd1* and *Pkdr1* and genetically modified mice with *Pkd1* knockout (KO)^11,12^. Qian et al. proposed a “two-hit” model in which a second somatic mutation, including loss of heterozygosity, is required in addition to intrinsic *PKD1* mutation to trigger renal cyst formation^13^. The gene dosage (haploinsufficiency) model hypothesizes that a reduction in *PKD1* expression levels below a critical threshold leads to renal cyst formation^14,15^. Although these rodent models have provided insights into ADPKD, the specific features of the models limit the ability to extrapolate the research outcomes to human patients. For example, mice carrying heterozygous *Pkd1* KO rarely exhibit renal cyst formation, and the manifestation of symptoms is limited in aged mice^14,16^. Thus, rodent models may not faithfully reproduce the features of human ADPKD symptoms, mainly because of species-specific differences in lifespan, metabolism, and anatomical and physiological characteristics^12^. Therefore, an animal model displaying ADPKD symptoms that closely resemble those of human patients is needed.

As pigs are considered to exhibit physiological and anatomical characteristics similar to those of humans, they have often been used to develop models for intractable hereditary human diseases^17,18^. Furthermore, recent progress in genome editing technology has enabled the generation of model pigs for monogenic diseases^19,20^. Genetically modified cloned pigs with heterozygous mutations in *PKD1*, including c.642_643insTGCT (*PKD1*^*TGCT ins/+*^) and c642_643insT (*PKD1*^*T ins/+*^), were produced using zinc finger nucleases targeting exon 5 of *PKD1*^21^. Unlike in the mouse model, this *PKD1* KO pig model displayed renal cyst formation at a young age. However, the pig model failed to fully resemble ADPKD in terms of cyst formation onset. Unlike human patients, who begin developing cysts during the fetal period, monoallelic *PKD1* KO pigs do not display renal cysts neonatally.

In the current study, we generated heterozygous *PKD1* KO cloned pigs harboring the unique mutation c.152_153insG (*PKD1*^*insG*^) in the first exon of *PKD1*. The resulting *PKD1*^*insG/+*^ cloned pigs displayed characteristics of an ADPKD model, including (i) neonatal renal cyst formation, (ii) progressive cyst development during animal growth, and (iii) sustained fertility after sexual maturation. Symptoms that appeared in the founder generation of the cloned animals were faithfully reproduced in descendants inheriting the mutant gene.

This paper provides a detailed approach for creating heterozygous *PKD1* KO (*PKD1*^*insG/+*^) mutant pigs using CRISPR-Cas9 gene-editing technology and somatic cell cloning. Phenotypic features of the mutant pigs, including the founder cloned animals and their progeny, are also discussed.

## Materials and methods

### Animal care and chemicals

All animal experiments, including genetic modifications performed in this study, were approved by the Institutional Animal Care and Use Committee of Meiji University (IACUC11-0003 and IACUC16-0008). All experiments were performed in accordance with the relevant guidelines and regulations. All chemicals were purchased from Sigma-Aldrich (St. Louis, MO, USA) unless otherwise indicated.

### Design and preparation of *PKD1* targeting guide RNA

Guide RNA (gRNA) was designed using an online CRISPR design tool (http://crispr.mit.edu/) to target the coding region of the first exon of porcine *PKD1*, which is on chromosome 3. The specificity of the designed gRNA was confirmed by searching for similar porcine sequences in GenBank. The gRNA target sequence (without the protospacer adjacent motif sequence) was as follows: 5′-GCCTGCCGCGTCAACTGCTC−3′. A synthetic DNA template consisting of the gRNA bound to a T7 promoter for in vitro transcription was purchased from Thermo Fisher Scientific (Waltham, MA, USA). The in vitro transcribed gRNA was prepared using the MEGAshortscript T7 transcription Kit (Thermo Fisher Scientific), purified with the MEGAClear kit (Thermo Fisher Scientific), and stored at −80 °C until use.

### Isolation of heterozygous *PKD1* mutant cells and culture conditions

A primary culture of porcine fetal fibroblast cells (male line) was used as the progenitor line to create *PKD1* heterozygous KO cells. The progenitor cells and their derivatives were seeded onto type I collagen-coated dishes or plates (Asahi Glass, Tokyo, Japan) and cultured in α-Minimum Essential Medium (Thermo Fisher Scientific) supplemented with 15% fetal bovine serum (Nichirei Bioscience, Tokyo, Japan), 100 U/mL penicillin, 100 µg/mL streptomycin, and 0.25 µg/mL amphotericin B (antibiotic-antimycotic solution; Thermo Fisher Scientific) in a humidified atmosphere containing 5% CO_2_ at 37 °C.

### Isolation of heterozygous *PKD1* mutant cells

The fetal fibroblasts were cultured to 70–90% confluence, washed twice with Dulbecco’s phosphate-buffered saline without calcium and magnesium (Thermo Fisher Scientific), and treated with 0.05% trypsin-EDTA (Thermo Fisher Scientific) to detach and collect the cells. Subsequently, 4 × 10^5^ cells were suspended in 40 μL of R buffer (supplied with the Neon Transfection System; Thermo Fisher Scientific). After 4 μL of purified gRNA (200 ng/μL) and 2 μL of *Cas9* mRNA (1 µg/µL; Thermo Fisher Scientific) were added, the cells were electroporated under the following conditions: pulse voltage, 1600 V; pulse width, 20 ms; pulse number, 1 (program #4). Following electroporation, the cells were cultured at 37 °C for 24 h in antibiotic-free medium and then for another 48 h in medium with antibiotics. After the second incubation (i.e., 72 h after electroporation), a limiting dilution was performed in five 96-well plates to obtain single cell-derived clones. Fourteen days after the limiting dilution step, cells from wells showing relatively high confluency (>50%) were selected and divided for further culture and mutation analysis, whereas cells from wells with low confluency (~50%) were not used in further experiments.

### Analysis of CRISPR-Cas9-induced mutations in nuclear donor cells

The target region of CRISPR-Cas9 was amplified by direct PCR from the cell clones using MightyAmp DNA polymerase (TaKaRa Bio, Shiga, Japan) and the corresponding primers (5′-TGTCGAGCCTGCAGCTGGATGC-3′ and 5′- CAGACAGGCCGCAGCTGCTGC-3′). Nested PCR was performed using PrimeSTAR HS DNA polymerase (TaKaRa Bio) and appropriate primers (5′-CAGACAGGCCGCAGCTGCTGC-3′ and 5′-AGTCCCACCGAGTGAGAAGTC-3′). The PCR fragment including the target region was examined using the sequencing primer 5′-CTTGCGCTGTCCTGACGATG-3′ and BigDye Terminator Cycle Sequencing Kit (Thermo Fisher Scientific) on an ABI PRISM 3130xl Genetic Analyzer (Applied Biosystems, Foster City, CA, USA).

### Off-target mutation analysis in nuclear donor cells

All potential off-target sites in the pig genome were predicted by an online CRISPR design tool (http://crispr.mit.edu/). The top 10 potential off-target sites were selected, and regions overlapping the sites were amplified by PCR using appropriate sets of primers (Supplementary Table S1) and cloned into TOPO vector according to the manufacturer’s instructions (Thermo Fisher Scientific). All positive colonies for each potential off-target site were analyzed by DNA sequencing.

### Somatic cell nuclear transfer and embryo transfer

Somatic cell nuclear transfer (SCNT) was performed as described previously with slight modifications^19,22^. Briefly, in vitro-matured oocytes containing the first polar body were enucleated via gentle aspiration of the polar body and adjacent cytoplasm using a beveled pipette in 10 mM HEPES-buffered Tyrode lactose medium containing 0.3% (w/v) polyvinylpyrrolidone, 0.1 μg/mL demecolcine, 5 μg/mL cytochalasin B, and 10% fetal bovine serum. Fibroblasts (cell clone #132) were used as nuclear donors following cell cycle synchronization via serum starvation for 2 days. A single donor cell was inserted into the perivitelline space of each enucleated oocyte. The donor cell-oocyte complexes were placed in a solution of 280 mM mannitol (Nacalai Tesque, Kyoto, Japan) containing 0.15 mM MgSO_4_, 0.01% (w/v) polyvinyl alcohol, and 0.5 mM HEPES (pH 7.2) and held between two electrode needles. Membrane fusion was induced with a somatic hybridizer (LF201; NEPA GENE, Chiba, Japan) by applying a single direct-current pulse (200 V/mm, 20 μs) and pre- and post-pulse alternating current field of 5 V at 1 MHz for 5 s. The reconstructed embryos were cultured in NCSU23 medium supplemented with 4 mg/mL bovine serum albumin for 1–1.5 h, followed by electrical activation performed as follows: the reconstructed embryos were washed twice in activation solution containing 280 mM mannitol, 0.05 mM CaCl_2_, 0.1 mM MgSO_4_, and 0.01% (w/v) polyvinyl alcohol; aligned between two wire electrodes (1.0 mm apart) of a fusion chamber slide filled with activation solution; and subjected to a single direct-current pulse of 150 V/mm for 100 μs using an electrical pulsing machine (Multiporator; Eppendorf, Hamburg, Germany). After activation, the reconstructed embryos were transferred into PZM-5 (porcine zygote medium 5) supplemented with 5 μg/mL cytochalasin B and 500 nM scriptaid for 3 h. The embryos were then transferred into PZM-5 supplemented with scriptaid only and cultured for another 12–14 h. These embryos were transferred to PZM-5 and maintained under a humidified atmosphere of 5% CO_2_, 5% O_2_, and 90% N_2_ at 38.5 °C. Beyond the morula stage, the embryos were cultured in PZM-5 supplemented with 10% fetal bovine serum. Crossbred (Large White/Landrace × Duroc) prepubertal gilts weighing 100–105 kg were used as recipients of the SCNT embryos. The gilts were given a single intramuscular injection of 1000 IU of equine chorionic gonadotropin to induce estrus. Ovulation was induced by intramuscular injection of 1500 IU of human chorionic gonadotropin human chorionic gonadotropin (Kawasaki Pharmaceutical, Kanagawa, Japan) administered at 66 h after equine chorionic gonadotropin injection. SCNT embryos that had been cultured for 5–6 days were surgically transferred into the oviducts of the recipients at approximately 146 h after human chorionic gonadotropin injection.

### Genotyping of *PKD1* heterozygous KO pigs by PCR-RFLP

The obtained piglets were genotyped using the polymerase chain reaction-restriction fragment length polymorphism (PCR-RFLP) method. First, genomic DNA was extracted from the tail biopsies of pigs using a DNeasy Blood and Tissue Kit (QIAGEN, Hilden, Germany) and subjected to nested PCR as described in the subsection “Analysis of CRISPR-Cas9-induced mutations in nuclear donor cells.” PCR products were digested with the restriction enzyme BsrI (New England Biolabs, Ipswich, MA, USA) at 65 °C for 1 h and then subjected to electrophoresis. The band profile was used to determine the zygosity of the sampled animal, as heterozygous *PKD1* mutant pigs were expected to display three bands: two from digestion of the mutant allele and one from digestion of the wild-type (WT) allele.

### Reverse transcription quantitative PCR

Total RNA was isolated from kidney specimens using RNeasy Plus Mini Kit with RNase-Free DNase Set (QIAGEN). cDNA was synthesized using SuperScript III First-Strand Synthesis Super Mix (Thermo Fisher Scientific). Quantitative PCR was performed using the StepOne Plus Real-Time PCR System (Thermo Fisher Scientific) and Premix Ex Taq (Probe qPCR) (Takara Bio) with the following primers and probes: Porcine *PKD1* TaqMan probe, 5′-CCGCGTCACCAGGAGCCTGGATGT-3′; *PKD1* forward primer (in exon 6), 5′-ACAGTCCCGCCGTCCAG-3′; *PKD1* reverse primer (in exon 7), 5′- CACAGCCGAGAAGCCGATC-3′, porcine *ACTB* (β-actin) TaqMan probe, 5′-CGGCTTTGCGGGCGACGATGCT-3′; *ACTB* forward primer, 5′-TGGATGACGATATTGCTGCGC-3′; *ACTB* reverse primer, 5′-GACACCAGGGCGTGATGG-3′. Both TaqMan probes were obtained from TaKaRa Bio. The ∆∆CT method was used to determine the relative expression normalized to *ACTB* expression. This experiment was performed three times, and the average results are reported.

### Diagnosis of renal cyst formation

The presence of renal cysts was confirmed by ultrasound examination using an HI VISION Avius (HITACHI, Tokyo, Japan) ultrasound system with an EUP-C175 Convex probe or FC1 (FUJIFILM SonoSite, Bothell, WA, USA) ultrasound device with a convex transducer C60xf (2–5 MHz). Pigs were intramuscularly injected with 1% mafoprazine mesylate (0.5 mg/kg, DS Pharma Animal Health Co., Ltd., Osaka, Japan), followed by intravenous injection of sodium thiopental (Nipro ES Pharma Co., Ltd., Osaka, Japan), and anesthesia was maintained via inhalation of isoflurane (DS Pharma Animal Health Co., Ltd.) while the pigs were examined. After anesthetization, cyst formation in the kidneys of heterozygous *PKD1* mutant pigs was examined ultrasonographically. Moreover, their longitudinal and transverse diameters were measured on the longitudinal plane. All ultrasonographic findings were evaluated by two nephrologists.

### Histological analysis

After *PKD1* heterozygous mutant pigs and age-matched WT pigs were sacrificed under anesthetization, the kidney tissues were dissected, fixed in 4% paraformaldehyde in phosphate-buffered saline without calcium and magnesium (Wako Pure Chemical Industries, Osaka, Japan), embedded in paraffin, sectioned, and subjected to Masson’s trichrome staining. For immunohistochemical analysis, the fixed sections were treated with a mouse anti-PC1 monoclonal (clone: 7e12) antibody (1:200 dilution) overnight at 4 °C. After removing excess antibody, the sections were incubated with Histofine Simple Stain MAX PO (MULTI) (Nichirei Bioscience) and DAB chromogen for 30 min at 25 °C. The slides were counterstained with hematoxylin and visualized under a BIOREVO BZ9000 microscope (Keyence, Osaka, Japan).

### Analysis of biochemical blood parameters

Under anesthetization, blood samples were collected in tubes containing heparin to determine the concentrations of blood creatinine (CRE), urea nitrogen (BUN), aspartate aminotransferase, alanine aminotransferase, and lactate dehydrogenase using a dry-chemistry analyzer (FUJI DRI-CHEM 7000, FUJIFILM Corporation, Tokyo, Japan).

### Statistical analysis

Statistical analyses of *PKD1* expression in the kidney were performed using SPSS Statistics 20.0 software (SPSS, Inc., Chicago, IL, USA). Student’s *t* test was used to compare differences between the WT and each *PKD1*^*insG/+*^ pig. A *p* value < 0.05 was considered to indicate statistically significant results.

## Results

### Design of gRNA and nuclear donor cell isolation

**Fig. 1:**
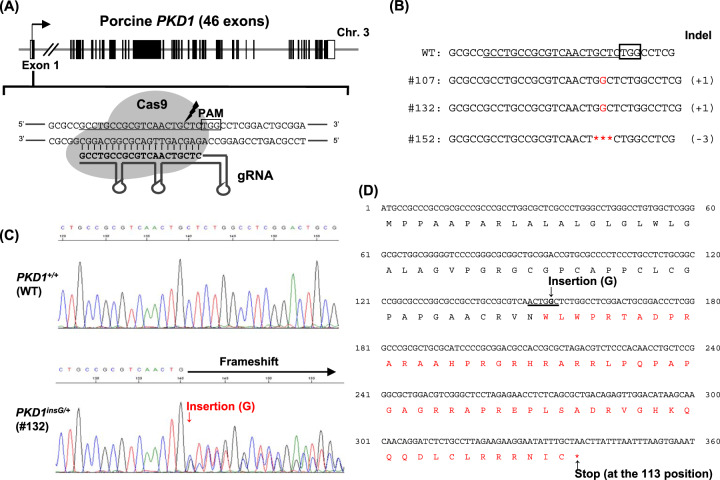
Design of CRISPR-Cas9 targeting of porcine *PKD1* and isolation of nuclear donor cells. **A** Schematic representation of CRISPR-Cas9 targeting site for porcine *PKD1* gene. The gRNA targeting sequences are underlined. The black box represents a protospacer adjacent motif (PAM) sequence. **B** CRISPR-Cas9-induced mutations in the isolated cell clones. The deletion mutation is indicated by asterisks. **C** Direct DNA sequence analysis of a *PKD1*^*insG/+*^ nuclear donor cell (clone #132). **D** Deduced amino acid sequence resulting from the induced mutation (*PKD1*^*insG*^) in cell clone #132. The amino acid sequences in red indicate nonsense amino acids. A 1-bp insertion of guanine nucleotide in porcine *PKD1* created a BsrI restriction enzyme site (underlined) and stop codon at amino acid residue 113.

In pigs, as in humans and mice, *PKD1* contains 46 exons^23^. We designed a gRNA targeting exon 1 of *PKD1* (Fig. 1A). Heterozygous *PKD1* mutant cells were generated by introducing gRNA and *Cas9* mRNA into male porcine fetal fibroblasts. Of the 163 cell clones obtained by limiting dilution, mutations were detected in three clones (1.8%, 3/163) (Fig. 1B). Cell clone #132, which showed no morphological aberrations and exhibited high proliferation, was chosen as the nuclear donor cell for SCNT. Cell clone #132 had a guanine nucleotide inserted between bases 152 and 153 of exon 1, generating mutant c.152_153insG (*PKD1*^*insG/+*^) with the resulting frameshift creating a premature stop codon (Fig. 1C–D). The mutated allele was expected to code for a truncated PC1 protein containing 112 amino acids (p.Cys51Trpfs*63). In addition to causing a frameshift, the addition of guanine nucleotide created a BsrI restriction endonuclease site, enabling RFLP genotyping for this mutation (Fig. 1D). DNA sequencing of cell clone #132 revealed no mutations in the top 10 potential off-target sites (Supplementary Table S1).

### Generation of heterozygous *PKD1* KO cloned pigs

**Fig. 2:**
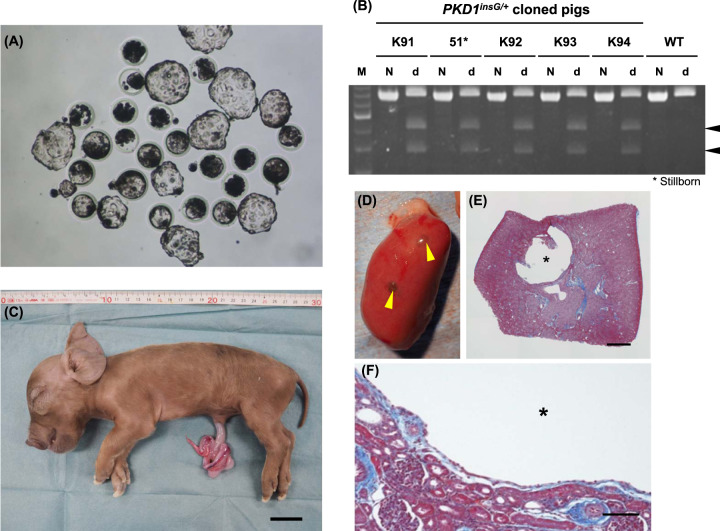
Generation of *PKD1*^*insG/+*^ cloned pigs by somatic cell nuclear transfer. **A** Cloned blastocysts generated using a *PKD1*^*insG/+*^ nuclear donor cell (clone #132), which were then transferred into recipient gilts. **B** Genotyping of four live *PKD1*^*insG/+*^ cloned pigs (K91, K92, K93, and K94) and one stillborn (No. 51) by PCR-RFLP analysis. One or three bands were obtained after BsrI digestion of the PCR amplicon from WT pigs and *PKD1*^*insG/+*^ pigs, respectively. Arrowheads indicate the digested fragments from the *PKD1*^*insG*^ mutant allele with a 1-bp insertion of a guanine nucleotide. M DNA Ladder, N non-digested, d BsrI digested. **C** Photograph of a stillborn *PKD1*^*insG/+*^ cloned pig (No. 51). The bar represents 3 cm. **D** Gross morphology of a kidney from the stillborn piglet. Yellow arrowheads indicate renal cysts. **E**, **F** Histological section of the kidney (Masson’s trichrome staining). Asterisks indicate a renal cyst. Bars = 1 mm (**E**) and 100 µm (**F**).

Using cell clone #132 as the nuclear donor, 330 cloned embryos carrying heterozygous *PKD1* KO *(PKD1*^*insG/+*^*)* were generated via SCNT. The in vitro development rate of these embryos was 68.8% (227/330) (Table 1), which is consistent with the results of our previous reports^19,24^. After transferring the 227 SCNT embryos into two recipient gilts, five heterozygous *PKD1* mutant cloned piglets were obtained, including one stillbirth (Table 1 and Fig. 2A–C). The cause of death of the stillborn offspring is unknown but may have occurred because of an accident during labor (Fig. 2C). Genotyping of the cloned pigs using PCR-RFLP analysis confirmed that they harbored the same *PKD1* mutation (c.152_153insG; *PKD1*^*insG/+*^) as the nuclear donor cells (Fig. 2B). The production efficiency of the *PKD1*^*insG/+*^ cloned pigs from the two gilts was 2.2% (5/227) (Table 1). These values were similar to those we reported previously^19,24–27^. Autopsy of one stillborn (No. 51) among the five offspring revealed renal cyst formation during the neonatal stage (Fig. 2C–E). These renal cysts were lined by epithelium (Fig. 2F).

**Table 1:** In vitro development of SCNT embryos and production of cloned pigs.

In vitro development of reconstructed SCNT embryos		
SCNT embryos reconstructed	330	
Normally cleaved embryos (day 2)	260 (78.8%)	
Blastocysts normally developed	227 (68.8%)	
Production of *PKD1*^*insG/+*^ cloned pigs		
Recipient No.	M174	M175
No. of embryos transferred	114	113
Cloned piglets obtained	1 (0.9%)	4^a^ (3.5%)

^a^1 stillborn pig included.

### Phenotypes of *PKD1*^*insG*/+^ mutant cloned pigs

**Fig. 3:**
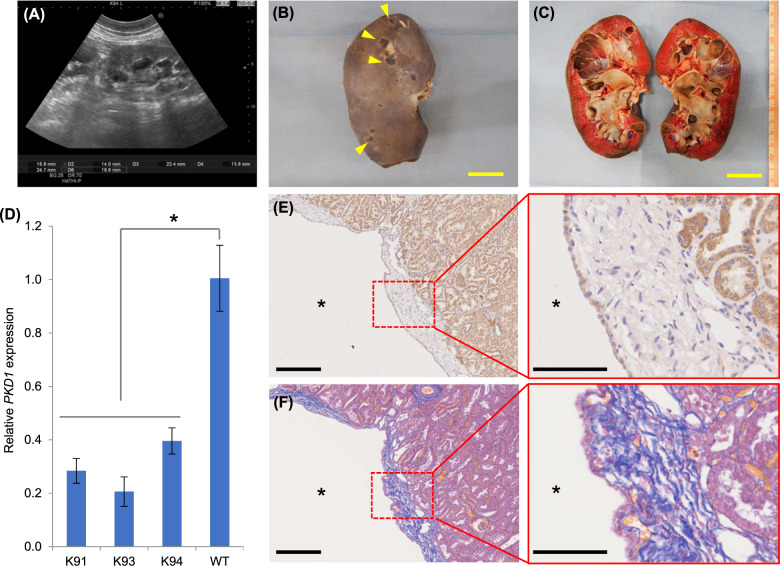
Phenotypes *of PKD1*^*insG/+*^ founder cloned pigs. **A** Ultrasound diagnostic images of the kidney from a *PKD1*^*insG/+*^ cloned pig (K94) at 5 months of age. Gross morphology (**B**) and coronal section (**C**) of a kidney from a *PKD1*^*insG/+*^ cloned pig. The yellow bar represents 3 cm. Yellow arrowheads indicate renal cysts. **D** Relative *PKD1* expression in three *PKD1*^*insG/+*^ cloned pigs evaluated using quantitative RT-PCR. The data are presented as the mean ± SD. *p < 0.05. **E**, **F** Histological analysis of a *PKD1*^*insG/+*^ founder cloned pig at 5 months of age. Kidney tissue sections with Masson’s trichrome staining (**E**) and immunostaining using a PKD1-specific antibody (**F**). Asterisks indicate renal cysts. The black bars represent 250 µm in the left panels and 100 µm in the right panels.

The four surviving *PKD1*^*insG/+*^ cloned pigs (K91–94) grew without any apparent or clinical abnormalities. Ultrasonography performed at 5 months of age confirmed cyst formation in the kidneys of all pigs (Fig. 3A and Supplementary Fig. S1A). Three of the pigs were euthanized and autopsied at 5 months (K93 and K94) or 8 months (K91) of age, and multiple macroscopic cysts were found in both kidneys of each animal (Fig. 3B, C and Supplementary Fig. S1B).

Reverse transcription quantitative PCR analysis of kidney specimens showed that *PKD1* expression levels in *PKD1*^*insG/+*^ cloned pigs were reduced by half compared to in WT pigs (Fig. 3D). These data indicate that RNA derived from the mutant allele was selectively degraded by nonsense-mediated decay because of the creation of a premature stop codon associated with the CRISPR-Cas9-induced mutation.

Histochemical analysis of kidney tissue from *PKD1*^*insG/+*^ cloned pigs (K91, K93, and K94) showed that the cyst walls were lined by squamous epithelium. Masson’s trichrome staining revealed fibrosis around the cyst wall (Fig. 3E). Immunostaining using an anti-PKD1 antibody revealed a PKD1-positive signal in the epithelial cells of the cyst wall (Fig. 3F).

**Fig. 4:**
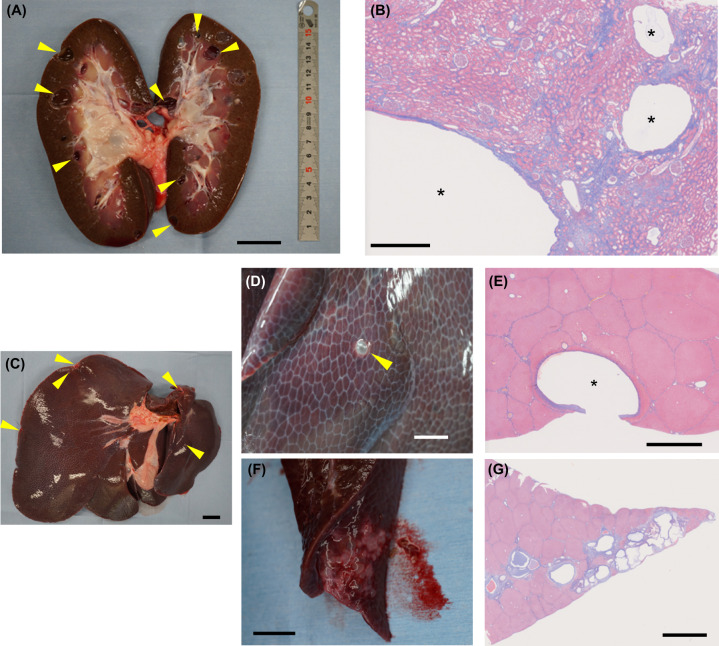
Renal and hepatic cysts in a *PKD1*^*insG/+*^ founder cloned pig at 36 months of age. **A** Coronal section of the kidney from a *PKD1*^*insG/+*^ clone (K92) at 36 months of age. The bar is 3 cm. **B** Kidney tissue sections with Masson’s trichrome staining. Asterisks indicate renal cysts. The bar is 1 mm. **C** Gross morphology of the liver from the cloned pig. Hepatic cysts are indicated by yellow arrowheads. The bar is 3 cm. **D**, **F** Macroscopic cysts and their histopathological evaluation using Masson’s trichrome staining (**E**, **G**). The asterisk indicates a hepatic cyst. The bars represent 2 mm (**D** and **F**), 2.5 mm (**E**), and 5 mm (**G**).

In addition to kidney cysts, multiple cysts were observed in the liver of an autopsied *PKD1*^*insG/+*^ cloned pig (K92) at 36 months of age, and fibrosis was observed around the liver cysts (Fig. 4). Serum CRE and BUN levels, as indicators of renal function, were 2.4 and 15.2 mg/dL, respectively. Aspartate aminotransferase, alanine aminotransferase, lactate dehydrogenase, as indicators of hepatocellular damage, were 68, 31, and 598 U/L, respectively. These levels were within the range of normal values for WT pigs (CRE, 1.0–2.7 mg/dL; BUN, 10–30 mg/dL; aspartate aminotransferase, 32–84 U/L; alanine aminotransferase, 31–58 U/L; lactate dehydrogenase, 380–630 U/L) ^28^.

### Reproduction of heterozygous *PKD1* mutant pigs and progeny phenotypes

Renal cyst formation was confirmed in all five produced *PKD1*^*insG/+*^ founder cloned pigs. We subsequently checked whether the *PKD1*^*insG*^ genotype introduced by CRISPR-Cas9 and its associated phenotype were transmitted to the next generation of animals. A total of 17 F1 offspring were produced by natural mating of a male founder clone pig (K92) and two WT female pigs (*PKD1*^*+/+*^; M237 and W239). Transmission of the mutant *PKD1*^*insG*^ was confirmed in 10 of the 17 progeny animals, demonstrating germline transmission of the mutant *PKD1* gene (Supplementary Table S2).

**Fig. 5:**
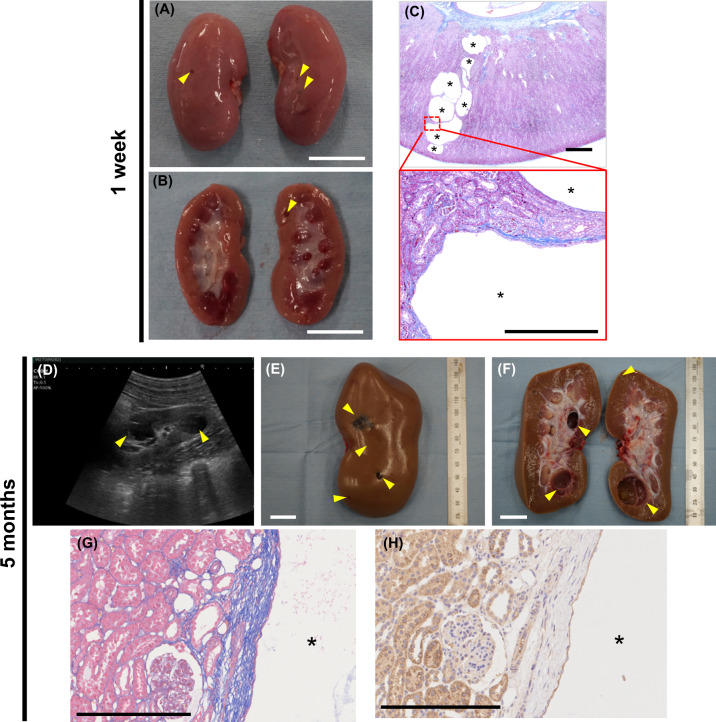
Phenotypes of *PKD1*^*insG/+*^ F1 progeny presenting early-onset renal cyst formation. *PKD1*^*insG/+*^ F1 progeny at 1 week of age (**A–C**) and 5 months of age (**D–H**). Gross morphology (**A**) and coronal section of the kidney (**B**) from *PKD1*^*insG/+*^ F1 progeny at 1 week of age. The arrowheads indicate renal cysts. The white bar represents 2 cm. **C** Kidney tissue sections with Masson’s trichrome staining of *PKD1*^*insG/+*^ F1 progeny. The black bars represent 1 mm in the upper panels and 250 µm in the lower panels. **D** Ultrasound diagnostic images of the kidney in *PKD1*^*insG/+*^ F1 progeny. Gross morphology (**E**) and coronal section (**F**) of the kidney from *PKD1*^*insG/+*^ F1 progeny (W282) at 5 months of age. The white bar represents 2 cm. Yellow arrowheads indicate renal cysts. **G** Kidney tissue sections of *PKD1*^*insG/+*^ F1 pigs with Masson’s trichrome staining and immunostaining using a PKD1-specific antibody (**H**). Asterisks indicate renal cysts. The bar represents 250 µm.

Autopsy of a newborn (1-week-old) *PKD1*^*insG/+*^ F1 animal born from one WT female pig (W239) revealed the presence of macroscopic cysts (Fig. 5A–C). Newborn *PKD1*^*insG/+*^ F1 pigs (*n* = 5) born from one WT female pig (M237) steadily grew similarly to WT pigs (Supplementary Fig. S2). Ultrasonography performed at 5 months of age revealed renal cyst formation in all five *PKD1*^*insG/+*^ F1 offspring evaluated (Fig. 5D–F, Supplementary Figs. S3 and S4). In addition, ultrasonography evaluation of one *PKD1*^*insG/+*^ F1 offspring (W322) at both 5 and 13 months revealed increased numbers of renal cysts as well as enlargement of the renal cysts from 5 months (1.42 × 1.26 cm) compared to that at 13 months (2.09 × 2.38 cm) (Supplementary Fig. S5A). Increased numbers of renal cysts over time were also observed in other *PKD1*^*insG/+*^ F1 offspring (Supplementary Fig. S5B).

Histochemical analysis of the resected kidneys of *PKD1*^*insG/+*^ mutant F1 progeny showed fibrosis around the cysts, similar to that of *PKD1*^*insG/+*^ mutant clones. Cyst-lining epithelial cells of the cyst walls showed mixed populations of cells with prominent and faint PKD1 signals (Fig. 5G, H).

We confirmed that the mutant allele was stably passed to the next generation (F2) of offspring by natural mating of a male *PKD1*^*insG/+*^ F1 pig (W279) that had reached sexual maturity with a WT female pig (#1730) (Supplementary Fig. S6A). Renal cysts were confirmed in all six *PKD1*^*insG/+*^ F2 newborns (3 days old) (Supplementary Fig. S6B). A very large number of cysts was observed in one of the F2 pigs (#1730-1), and fibrosis was observed around the cysts (Supplementary Fig. S7A–C). Furthermore, PKD1-positive signals were observed in cyst-lining epithelial cells (Supplementary Fig. S7D). These results show that the development of renal cysts in *PKD1*^*insG/+*^ mutant pigs during the neonatal period was not only a feature of the cloned founder but also common to the F1 and F2 progeny.

Serum CRE and serum BUN levels of F1 progeny at 24 months of age were 2.4–2.5 mg/dL (*n* = 3) and 5.1–13.0 mg/dL (*n* = 3), respectively. Both the CRE and BUN levels in the F1 progeny were within the normal range of those in the WT pigs. CRE and BUN levels remained in the normal range (2.2 and 17.7 mg/dL, respectively) in an F1 progeny pig that had been raised until 53 months of age. No clinical abnormalities were detected in the renal function of *PKD1*^*insG/+*^ pigs, even after 4 years of age.

*Pkd1* homozygous KO mice are known to exhibit embryonic or prenatal lethality^16^. Mating of two F1 *PKD1*^*insG/+*^ boars (W279 and W280) with two gilts (W273 and W322) with the same mutation gave rise to a total of 19 F2 offspring including only *PKD1*^*insG/+*^ and *PKD1*^*+/+*^ pigs, with no homozygous *PKD1*-KO pigs (*PKD1*^*insG/insG*^) born (Supplementary Table S3). In addition, genotyping of 39-day-old fetuses (*n* = 6) produced by mating F1 *PKD1*^*insG/+*^ animals did not reveal homozygous *PKD1*-KO fetuses (Supplementary Table S3). These results indicate early embryonic lethality of the homozygous *PKD1*-KO in the pig.

## Discussion

In heterozygous *Pkd1-*KO mice, renal cyst formation is very rare, even at an old age^29,30^. This may reflect the limitations of rodent models for studying this disease, as rodents have a short lifespan and markedly different anatomy and physiology compared to humans. In contrast, the *PKD1*^*insG/+*^ pigs produced in this study (Supplementary Table S4) with a mutation introduced into exon 1 of *PKD1* (c.152_153insG) by CRISPR-Cas9 exhibited a similar phenotype as human patients with ADPKD, including high penetrance of renal cyst formation. We confirmed that the mutant allele was transmitted following Mendelian inheritance to progeny offspring. Furthermore, the progeny pigs faithfully reproduced the renal cystic phenotype, which is the major symptom of ADPKD.

Patients with ADPKD generally show slow progression of renal cyst formation, and most patients are clinically asymptomatic until their 4th to 5th decade of life^1,4,5^. However, renal cyst formation in patients with ADPKD occurs starting during the fetal period, with cysts progressively increasing in number and size with age^31–33^. In the heterozygous *PKD1*-KO minipigs reported previously^21^, no macroscopic or microscopic cysts were observed in the neonatal stage. In contrast, *PKD1*^*insG/+*^ pigs produced in the current study exhibited macroscopic cyst formation during the neonatal stage, similar to in human patients with ADPKD. These human-like cystogenic dynamics, combined with progressive renal cyst formation in conjunction with age, may be a hallmark of *PKD1*^*insG/+*^ pigs.

The difference in the timing of renal cyst formation between the current study and that reported by He et al.^21^ may be related to the differences in the type of mutation of the causative gene. Various types of *PKD1* mutations have been identified in patients with ADPKD, including missense, nonsense, in-frame deletion/insertion, and aberrant splicing mutations^34,35^. It has been reported that over 70% of detected mutations are unique^36^. Together, our findings suggest that diverse causal mutations result in ADPKD symptoms in pigs.

In contrast, differences in pathological conditions within a family of patients with the same mutation and among siblings have been reported^37^. The genetic background of *PKD1*^*insG/+*^ cloned pigs (K91–K94) obtained in this study is identical. The degree of cyst formation exhibited by these individuals varied. Although gene mutation is among the major determinants of disease severity, various factors are involved in cyst formation in a complicated manner in ADPKD. Our *PKD1*^*insG/+*^ cloned pig data suggest the presence of modifiers that differ from the genetic background of the individual. Regarding the difference in the pathophysiology of K94 and K92 in *PKD1*^*insG/+*^ cloned pigs, we have not yet identified the cause of individual differences. Differences in genetic background, nongenetic factors including epigenetic factors, and microRNAs are thought to have caused this discrepancy^38–41^. The development of ADPKD model pigs with various mutation types will provide diverse cystogenic dynamics to give a detailed understanding of the pathophysiology of ADPKD and improve the development of treatment methods.

A two-hit theory has been proposed as a mechanism of cyst formation in patients with ADPKD^13^. According to this concept, the etiology of ADPKD involves a heterozygously inherited mutation and somatic mutation in the other normal *PKD1* allele, resulting in the complete loss of function of *PKD1*, ultimately leading to renal cyst formation. In this theory, the proliferation of cystic epithelial cells is explained by the monoclonal proliferation of constituent cells in the nephron harboring the two genetic hits^13^. However, Nishio et al.^42^ described that renal cyst formation cannot be ascribed only to monoclonal proliferation of cystic epithelial cells.

In our *PKD1*^*insG/+*^ pigs, PKD1-positive signals were observed in many epithelial cells forming the renal cyst walls. Similarly, cysts in many patients with ADPKD have been reported to contain PKD1-positive cells^43,44^. This finding is consistent with the low frequency of cells displaying complete loss of *PKD1* function in individual renal cysts of patients with ADPKD. The two-hit theory can explain the slow onset of ADPKD and focal cyst formation but fails to adequately explain the full extent of the pathological manifestation of ADPKD^45,46^. Further study is required, such as investigating the mechanism of haploinsufficiency proposed in recent years^15,47^.

In addition to renal cyst formation during the neonatal stage, *PKD1*^*insG/+*^ pigs exhibited several characteristics similar to those of human ADPKD. First, the number of cysts that developed in the kidneys was similar to that in human patients with ADPKD. The diagnostic criteria for human ADPKD are met if a patient under 30 years of age has more than two or three unilateral or bilateral kidney cysts^48,49^. All young adult to adult *PKD1*^*insG/+*^ pigs evaluated presented with an adequate number of cysts to meet the diagnostic criteria of ADPKD in human patients.

The second similarity was interstitial fibrosis. Various degrees of interstitial fibrosis are observed around expanding renal cysts in patients with ADPKD^50^. Fibrosis was also observed around the renal cysts of *PKD1*^*insG/+*^ pigs, demonstrating a pathological presentation similar to that observed in humans.

Similarities or differences between *PKD1*^*insG/+*^ and human patients with ADPKD in the mechanisms of renal cyst development require further investigation. In human ADPKD, renal cysts derived from the collecting duct are thought to develop consistently, whereas renal cysts can originally arise from all segments of the nephron^51–53^. The origin of cyst formation in *PKD1*^*insG/+*^ pigs should be identified in further studies.

In many cases of ADPKD, renal function remains undiminished until midlife. It has been reported that in patients with progressive renal enlargement, their nephrons undergo compensatory hyperfiltration to maintain the glomerular filtration rate for decades, which continues until more than half of the functioning parenchyma is destroyed. Furthermore, CRE levels are maintained within the normal range^54^. The timing of clinical symptom onset in *PKD1*^*insG/+*^ pigs was also similar to that of human patients with ADPKD. No clinical renal hypofunction was detected in either the 36-month-old *PKD1*^*insG/+*^ founder clone or 53-month-old *PKD1*^*insG/+*^ F1 pig analyzed in the current study. The age of these pigs, considering the average lifespan of livestock pigs as approximately 20 years^55^, would correspond to a premature age in humans. Accordingly, we considered that *PKD1*^*insG/+*^ pigs also had an asymptomatic period during the first half of life, similar to that in humans with ADPKD. Further long-term studies are needed to clarify disease progression and disease latency in *PKD1*^*insG/+*^ pigs.

Recently, ADPKD models with mutations in *PKD1* of cynomolgus monkeys were created by zygote injection of CRISPR-Cas9^56^. The resulting *PKD1-*mutated monkeys exhibit a phenotype similar to our heterozygous *PKD1-*KO pigs. In fact, heterozygous *PKD1* monkeys show few renal cysts perinatally, and many live monkeys with *PKD1* mutations present only mild cyst formation. It has also been reported that there are no abnormalities in CRE or urinary urea nitrogen levels at 6 or 12 months. Therefore, *PKD1*-mutated monkeys are also thought to exhibit asymptomatic stages, similar to in our current *PKD1*^*insG/+*^ pigs.

Co-development of liver cysts, in addition to renal cyst formation, is known to be a common phenotype of patients with ADPKD^57^. The onset dynamics of liver cysts in *PKD1*^*insG/+*^ pigs is not clear, although we observed liver cyst formation in one of the founder clones at 36 months of age. Autosomal recessive polycystic kidney disease (ARPKD) due to *PKHD1* mutation is characterized by severe hepatic fibrosis with bile duct dysplasia and intrahepatic periportal fibrosis. In addition, congenital hepatic fibrosis is a complication associated with ARPKD^58^. The pathophysiology of the ADPKD model introduced with the *PKD1* mutation in this study differs from that of the fibrocystic liver phenotype in ARPKD. Other extrarenal manifestations of ADPKD include cyst formation in the pancreas and testes, hypertension, valvular heart disease, cerebral aneurysm, and diverticulosis^5^. Extrarenal manifestations in *PKD1*^*insG/+*^ pigs have not yet been determined. Pigs show greater fecundity than monkeys, and large animals have relatively early sexual maturation (pigs, 6–8 months; monkeys, 3–4 years). The normal reproductive ability of *PKD1*^*insG/+*^ pigs will allow for further research.

In conclusion, *PKD1*^*insG/+*^ pigs showing 100% penetrance of a cystic kidney phenotype have many symptomatic similarities to patients with ADPKD caused by heterozygous mutation of *PKD1*. Similarities include the formation of macroscopic renal cysts during the neonatal stage, number and cystogenic dynamics of renal cysts formed, pathological features such as interstitial fibrosis, and presence of an asymptomatic prematuration stage. ADPKD is treated clinically as an adult-onset disease; however, intervention starting at an earlier stage is needed^59^. *PKD1*^*insG/+*^ pigs can be used to study early intervention in pediatric patients with ADPKD, verify the effects of prophylactic treatment, and test long-term treatments that cannot be performed using rodent models.

## Supplementary information

Supple_Figs_Tables

## Data Availability

The datasets generated during and/or analyzed during the current study are available from the corresponding author on reasonable request.
