# Supplementary material for: Generation of heterozygous PKD1 mutant pigs exhibiting early-onset renal cyst formation
Source: Lab Invest. 2022 Jan 3;102(5):560–9. doi: 10.1038/s41374-021-00717-z (PMC9042704; doi:10.1038/s41374-021-00717-z)
Supplement: Supplementary file 1 — Supple_Figs_Tables [file 41374_2021_717_MOESM1_ESM.pptx]

## Slide 1
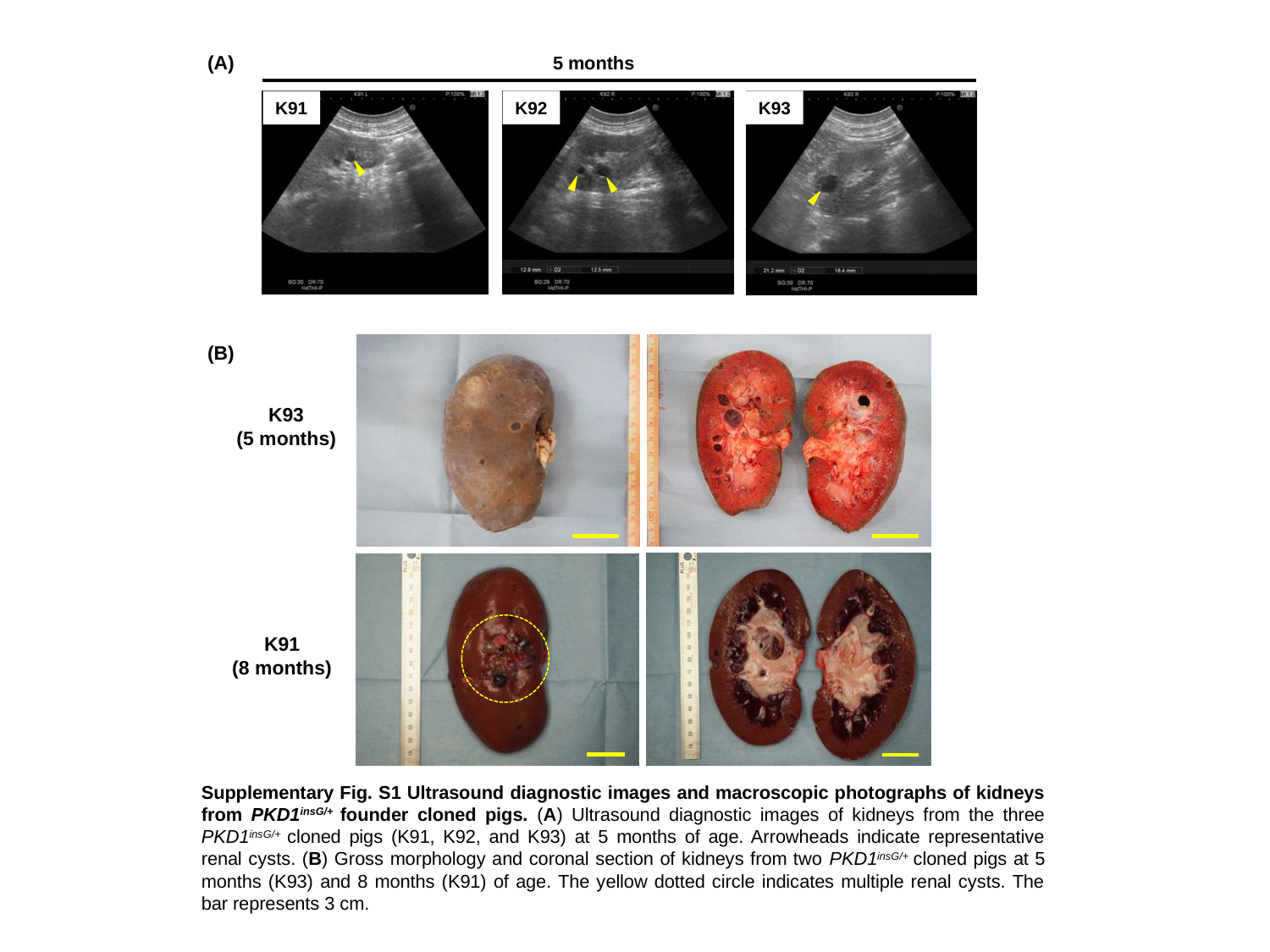

(A)
5 months
K93
K92
K91
(B)
K93
(5 months)
K91
(8 months)
Supplementary Fig. S1 Ultrasound diagnostic images and macroscopic photographs of kidneys from PKD1insG/+ founder cloned pigs. (A) Ultrasound diagnostic images of kidneys from the three PKD1insG/+ cloned pigs (K91, K92, and K93) at 5 months of age. Arrowheads indicate representative renal cysts. (B) Gross morphology and coronal section of kidneys from two PKD1insG/+ cloned pigs at 5 months (K93) and 8 months (K91) of age. The yellow dotted circle indicates multiple renal cysts. The bar represents 3 cm.

## Slide 2
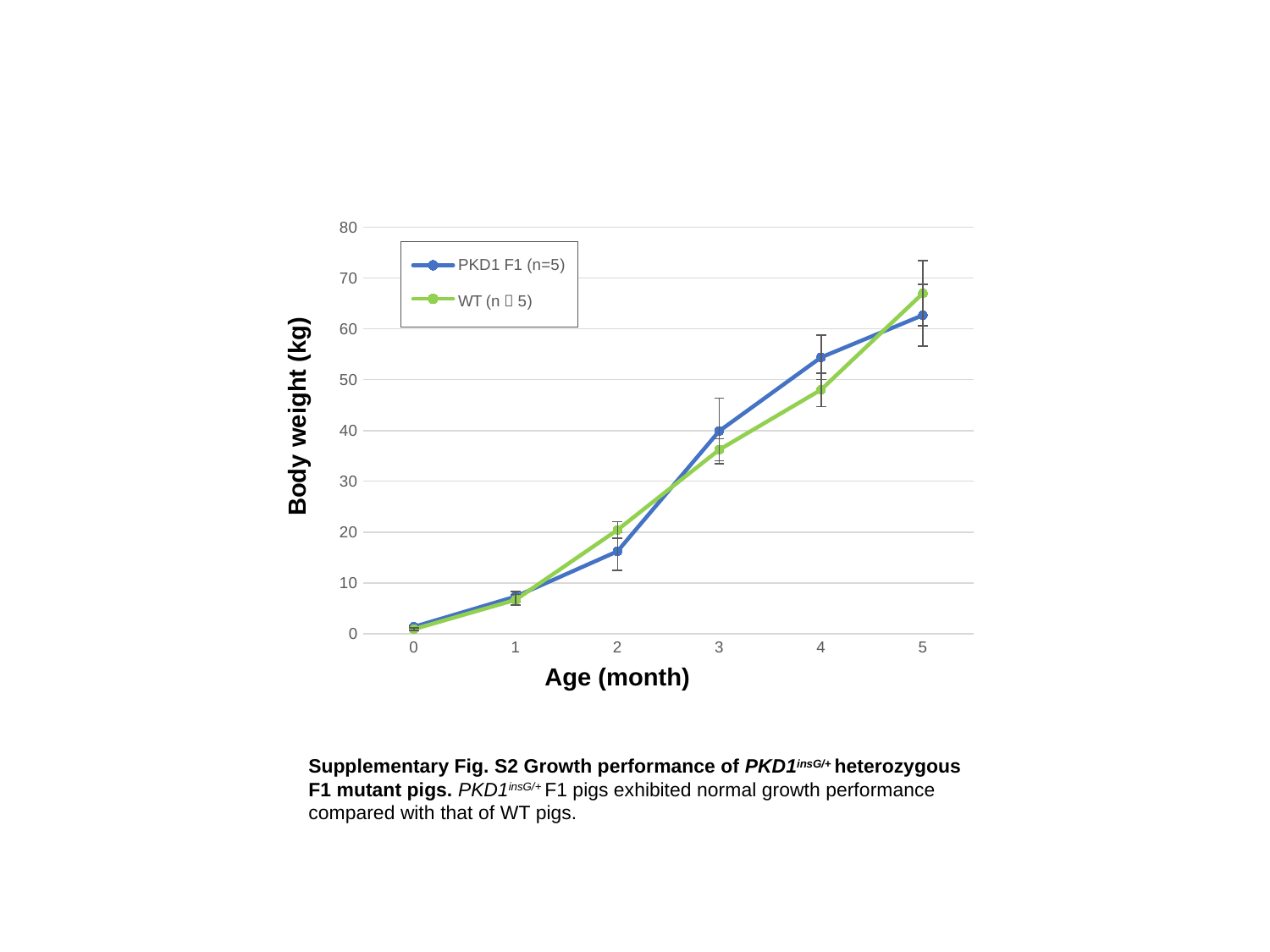

### Chart
| Category | PKD1 F1 (n=5) | WT (n＝5) |
|---|---|---|
| 0 | 1.345 | 0.8778 |
| 1 | 7.3 | 6.65975 |
| 2 | 16.2 | 20.419999999999998 |
| 3 | 39.9 | 36.220000000000006 |
| 4 | 54.4 | 48.02 |
| 5 | 62.7 | 67.0 |Body weight (kg)
Age (month)
Supplementary Fig. S2 Growth performance of PKD1insG/+ heterozygous F1 mutant pigs. PKD1insG/+ F1 pigs exhibited normal growth performance compared with that of WT pigs.

## Slide 3
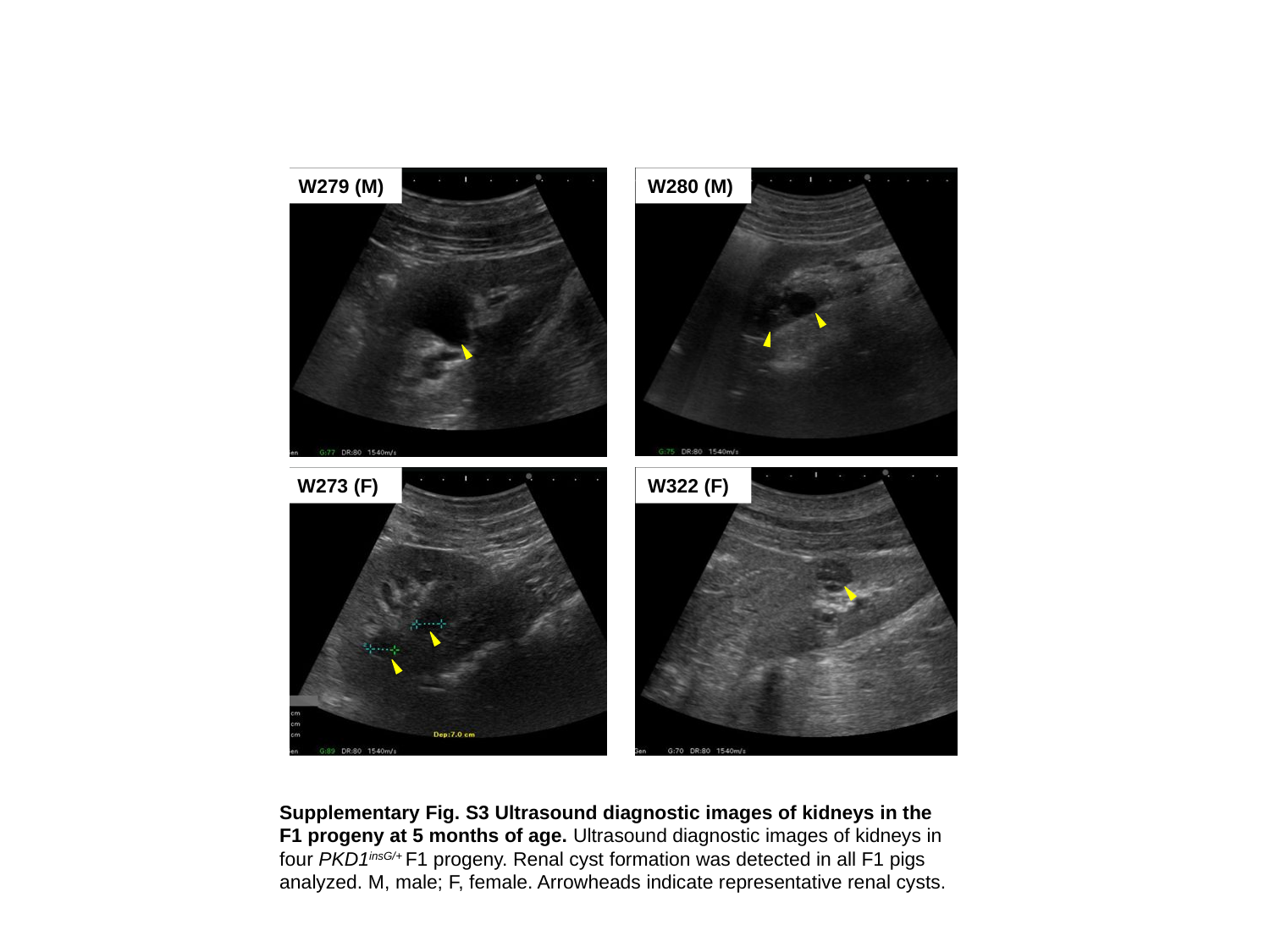

W280 (M)
W279 (M)
W273 (F)
W322 (F)
Supplementary Fig. S3 Ultrasound diagnostic images of kidneys in the F1 progeny at 5 months of age. Ultrasound diagnostic images of kidneys in four PKD1insG/+ F1 progeny. Renal cyst formation was detected in all F1 pigs analyzed. M, male; F, female. Arrowheads indicate representative renal cysts.

## Slide 4
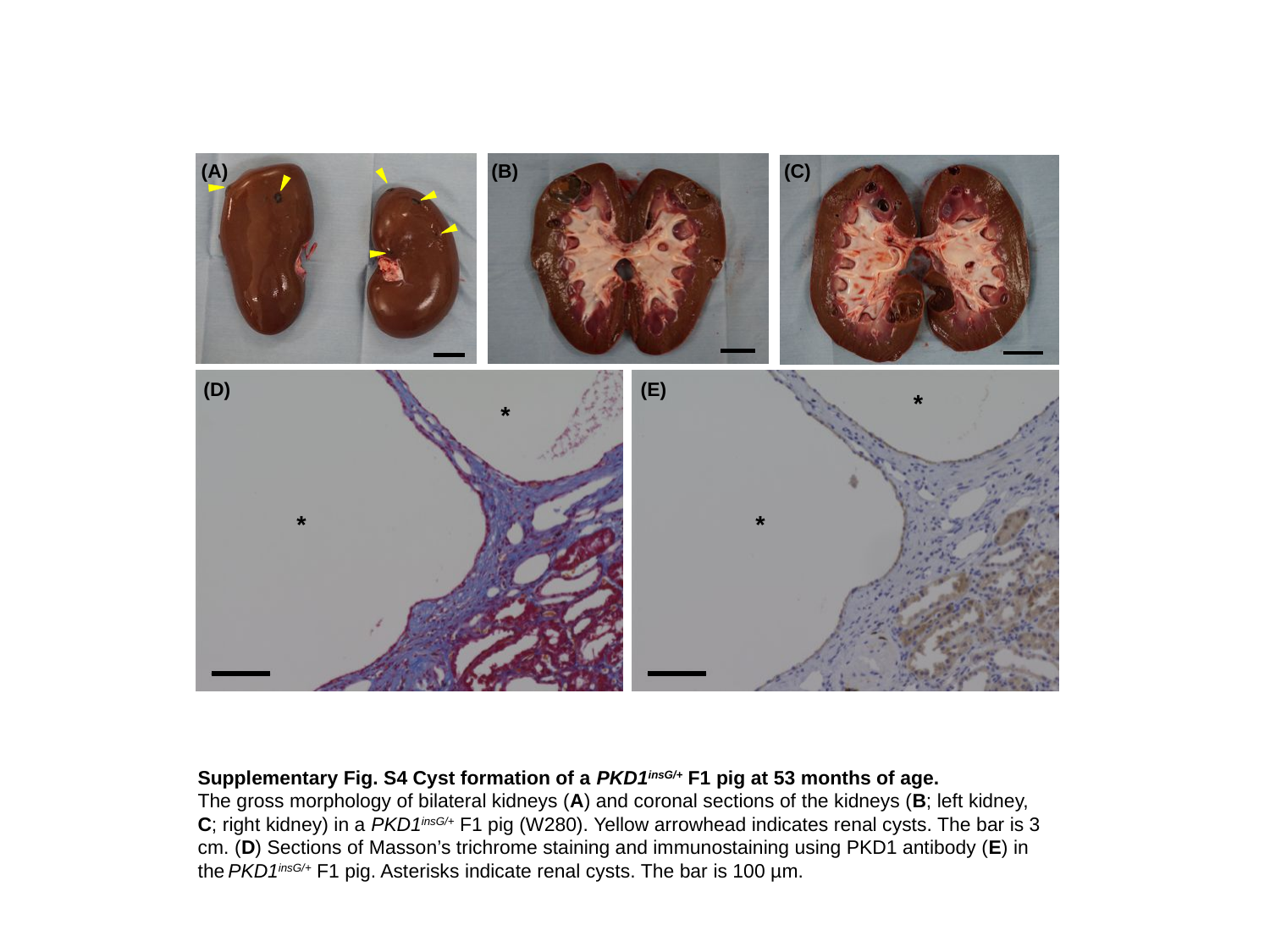

(A)
(B)
(C)
*
*
*
*
(D)
(E)
Supplementary Fig. S4 Cyst formation of a PKD1insG/+ F1 pig at 53 months of age.
The gross morphology of bilateral kidneys (A) and coronal sections of the kidneys (B; left kidney, C; right kidney) in a PKD1insG/+ F1 pig (W280). Yellow arrowhead indicates renal cysts. The bar is 3 cm. (D) Sections of Masson’s trichrome staining and immunostaining using PKD1 antibody (E) in the PKD1insG/+ F1 pig. Asterisks indicate renal cysts. The bar is 100 µm.

## Slide 5
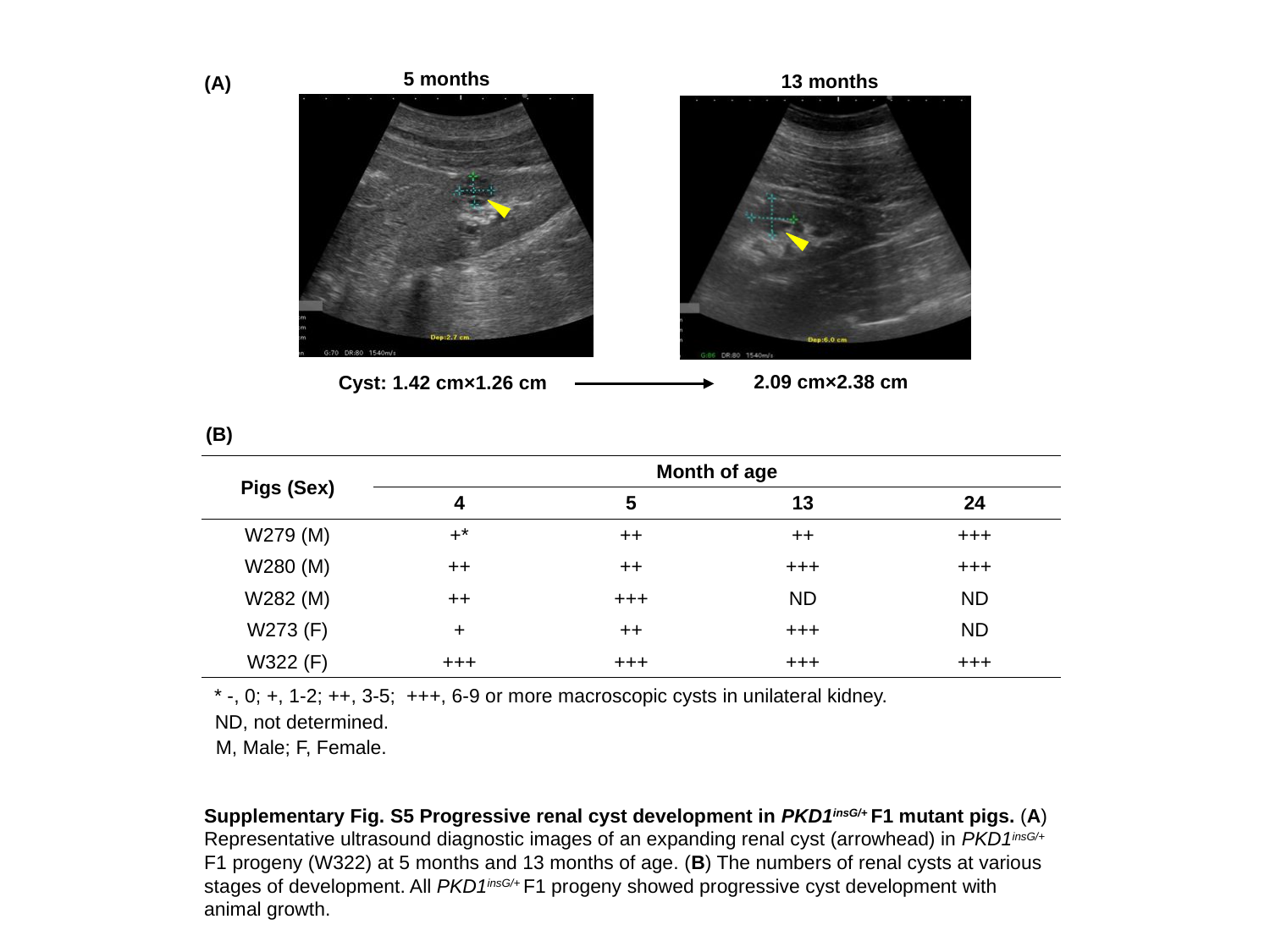

5 months
13 months
(A)
2.09 cm×2.38 cm
Cyst: 1.42 cm×1.26 cm
(B)
| Pigs (Sex) | Month of age | | | |
| --- | --- | --- | --- | --- |
| | 4 | 5 | 13 | 24 |
| W279 (M) | +\* | ++ | ++ | +++ |
| W280 (M) | ++ | ++ | +++ | +++ |
| W282 (M) | ++ | +++ | ND | ND |
| W273 (F) | + | ++ | +++ | ND |
| W322 (F) | +++ | +++ | +++ | +++ |
* -, 0; +, 1-2; ++, 3-5; +++, 6-9 or more macroscopic cysts in unilateral kidney.
ND, not determined.
M, Male; F, Female.
Supplementary Fig. S5 Progressive renal cyst development in PKD1insG/+ F1 mutant pigs. (A) Representative ultrasound diagnostic images of an expanding renal cyst (arrowhead) in PKD1insG/+ F1 progeny (W322) at 5 months and 13 months of age. (B) The numbers of renal cysts at various stages of development. All PKD1insG/+ F1 progeny showed progressive cyst development with animal growth.

## Slide 6
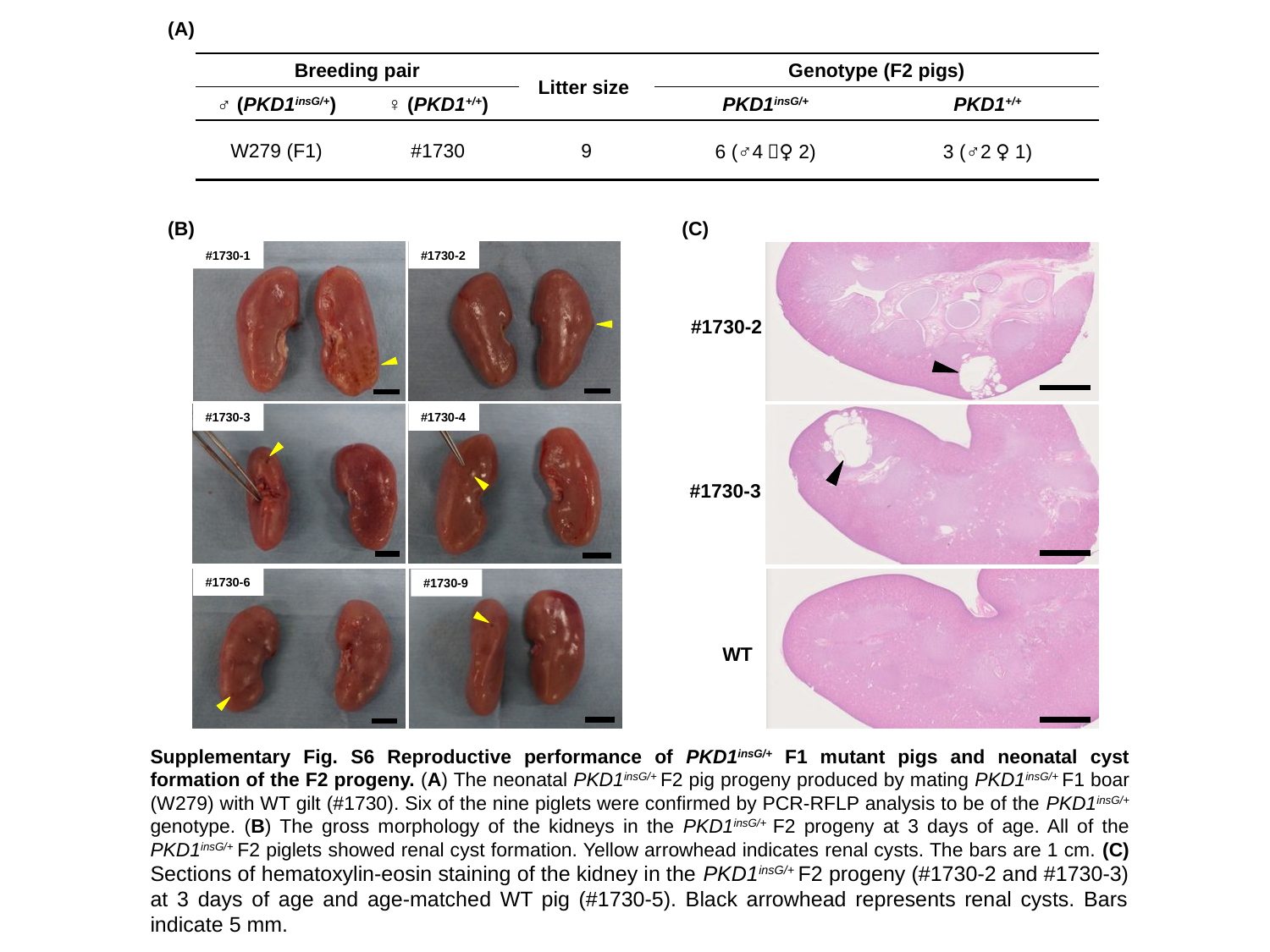

(A)
| Breeding pair | | Litter size | Genotype (F2 pigs) | |
| --- | --- | --- | --- | --- |
| ♂ (PKD1insG/+) | ♀ (PKD1+/+) | | PKD1insG/+ | PKD1+/+ |
| W279 (F1) | #1730 | 9 | 6 (♂4：♀2) | 3 (♂2：♀1) |
(B)
(C)
#1730-1
#1730-2
#1730-4
#1730-3
#1730-6
#1730-9
#1730-2
#1730-3
WT
Supplementary Fig. S6 Reproductive performance of PKD1insG/+ F1 mutant pigs and neonatal cyst formation of the F2 progeny. (A) The neonatal PKD1insG/+ F2 pig progeny produced by mating PKD1insG/+ F1 boar (W279) with WT gilt (#1730). Six of the nine piglets were confirmed by PCR-RFLP analysis to be of the PKD1insG/+ genotype. (B) The gross morphology of the kidneys in the PKD1insG/+ F2 progeny at 3 days of age. All of the PKD1insG/+ F2 piglets showed renal cyst formation. Yellow arrowhead indicates renal cysts. The bars are 1 cm. (C) Sections of hematoxylin-eosin staining of the kidney in the PKD1insG/+ F2 progeny (#1730-2 and #1730-3) at 3 days of age and age-matched WT pig (#1730-5). Black arrowhead represents renal cysts. Bars indicate 5 mm.

## Slide 7
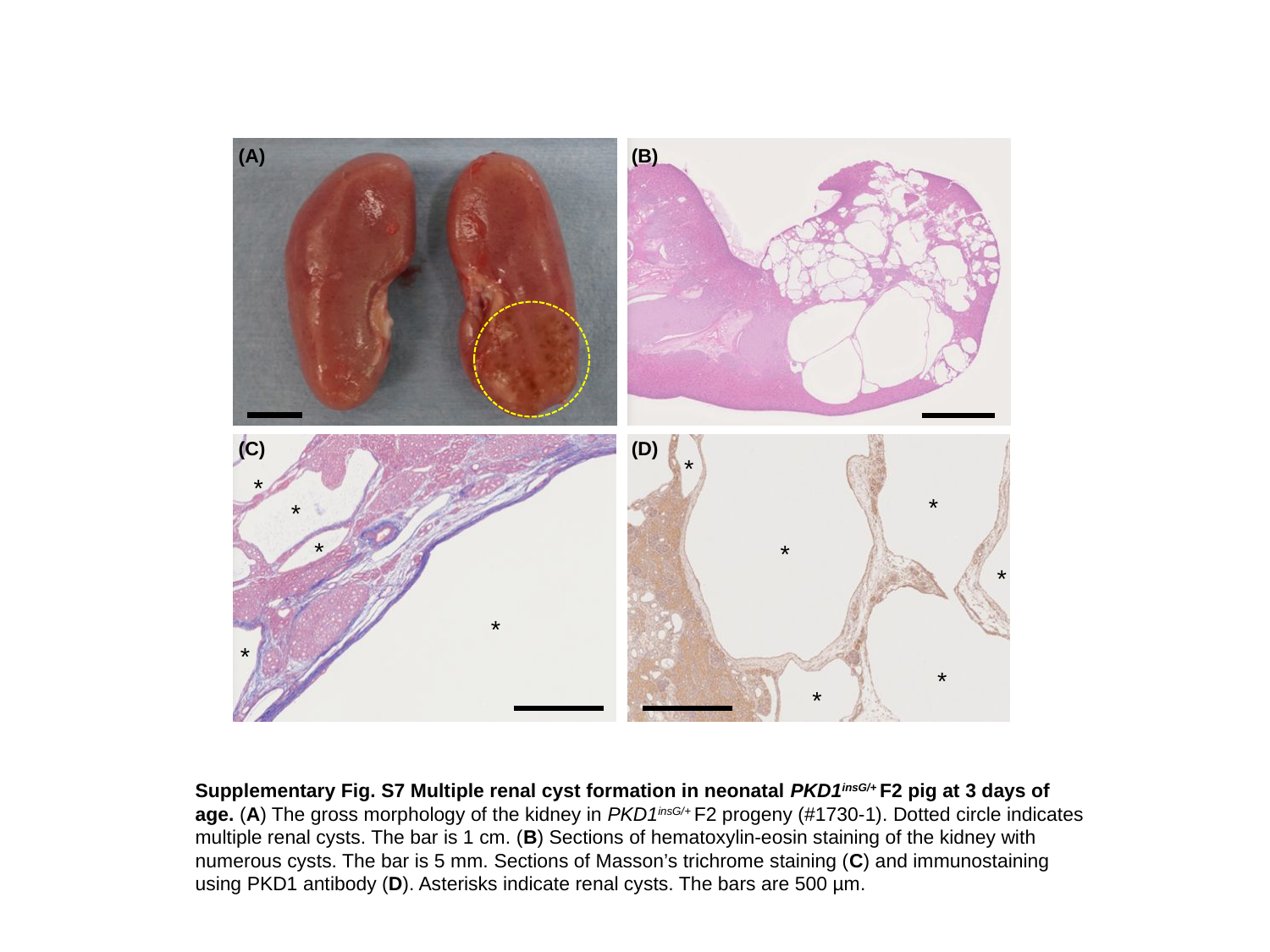

(A)
(B)
(C)
(D)
*
*
*
*
*
*
*
*
*
*
*
Supplementary Fig. S7 Multiple renal cyst formation in neonatal PKD1insG/+ F2 pig at 3 days of age. (A) The gross morphology of the kidney in PKD1insG/+ F2 progeny (#1730-1). Dotted circle indicates multiple renal cysts. The bar is 1 cm. (B) Sections of hematoxylin-eosin staining of the kidney with numerous cysts. The bar is 5 mm. Sections of Masson’s trichrome staining (C) and immunostaining using PKD1 antibody (D). Asterisks indicate renal cysts. The bars are 500 µm.

## Slide 8
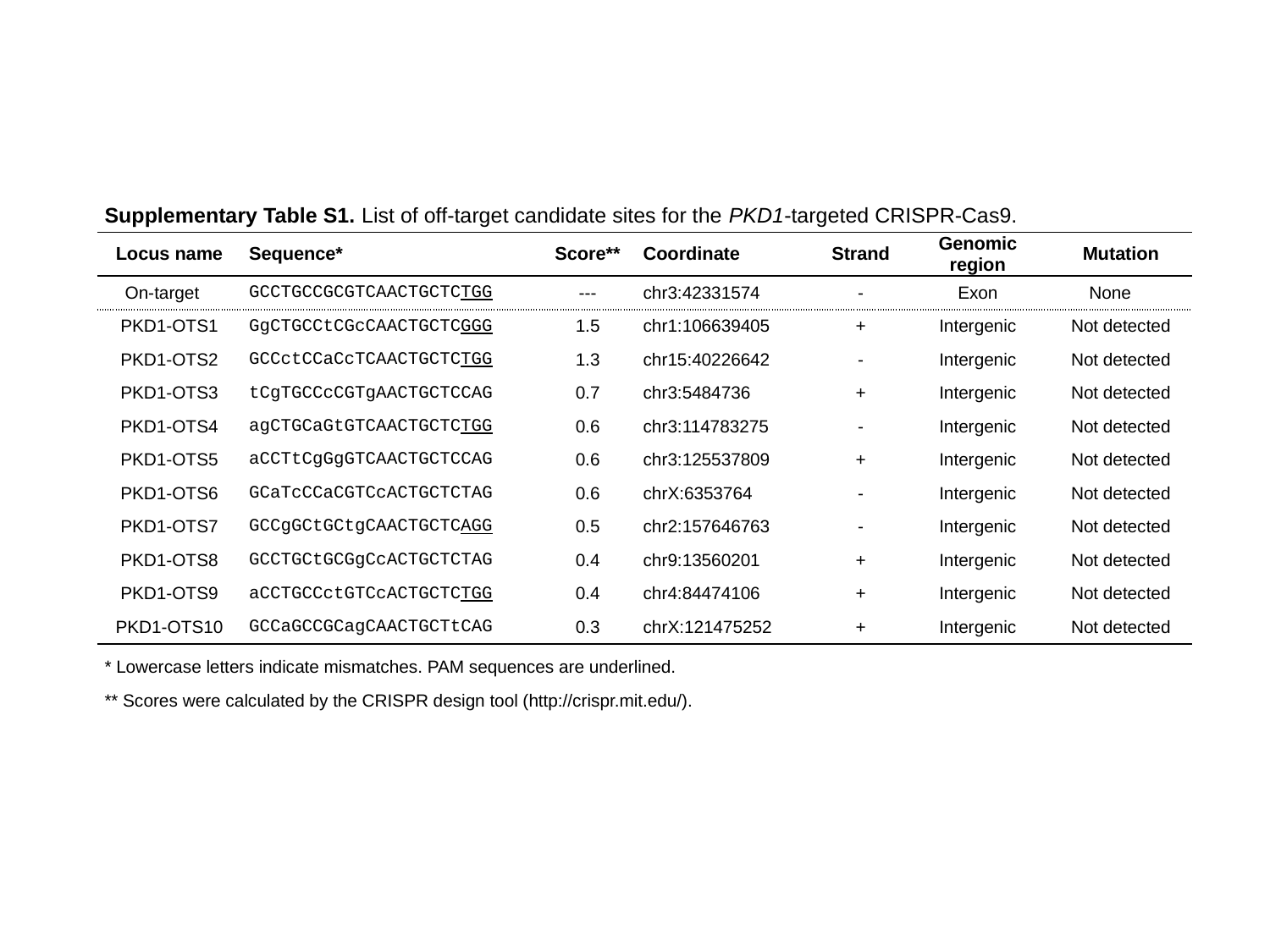

| Supplementary Table S1. List of off-target candidate sites for the PKD1-targeted CRISPR-Cas9. | | | | | | |
| --- | --- | --- | --- | --- | --- | --- |
| Locus name | Sequence\* | Score\*\* | Coordinate | Strand | Genomic region | Mutation |
| On-target | GCCTGCCGCGTCAACTGCTCTGG | --- | chr3:42331574 | - | Exon | None |
| PKD1-OTS1 | GgCTGCCtCGcCAACTGCTCGGG | 1.5 | chr1:106639405 | + | Intergenic | Not detected |
| PKD1-OTS2 | GCCctCCaCcTCAACTGCTCTGG | 1.3 | chr15:40226642 | - | Intergenic | Not detected |
| PKD1-OTS3 | tCgTGCCcCGTgAACTGCTCCAG | 0.7 | chr3:5484736 | + | Intergenic | Not detected |
| PKD1-OTS4 | agCTGCaGtGTCAACTGCTCTGG | 0.6 | chr3:114783275 | - | Intergenic | Not detected |
| PKD1-OTS5 | aCCTtCgGgGTCAACTGCTCCAG | 0.6 | chr3:125537809 | + | Intergenic | Not detected |
| PKD1-OTS6 | GCaTcCCaCGTCcACTGCTCTAG | 0.6 | chrX:6353764 | - | Intergenic | Not detected |
| PKD1-OTS7 | GCCgGCtGCtgCAACTGCTCAGG | 0.5 | chr2:157646763 | - | Intergenic | Not detected |
| PKD1-OTS8 | GCCTGCtGCGgCcACTGCTCTAG | 0.4 | chr9:13560201 | + | Intergenic | Not detected |
| PKD1-OTS9 | aCCTGCCctGTCcACTGCTCTGG | 0.4 | chr4:84474106 | + | Intergenic | Not detected |
| PKD1-OTS10 | GCCaGCCGCagCAACTGCTtCAG | 0.3 | chrX:121475252 | + | Intergenic | Not detected |
| \* Lowercase letters indicate mismatches. PAM sequences are underlined. | | | | | | |
| \*\* Scores were calculated by the CRISPR design tool (http://crispr.mit.edu/). | | | | | | |

## Slide 9
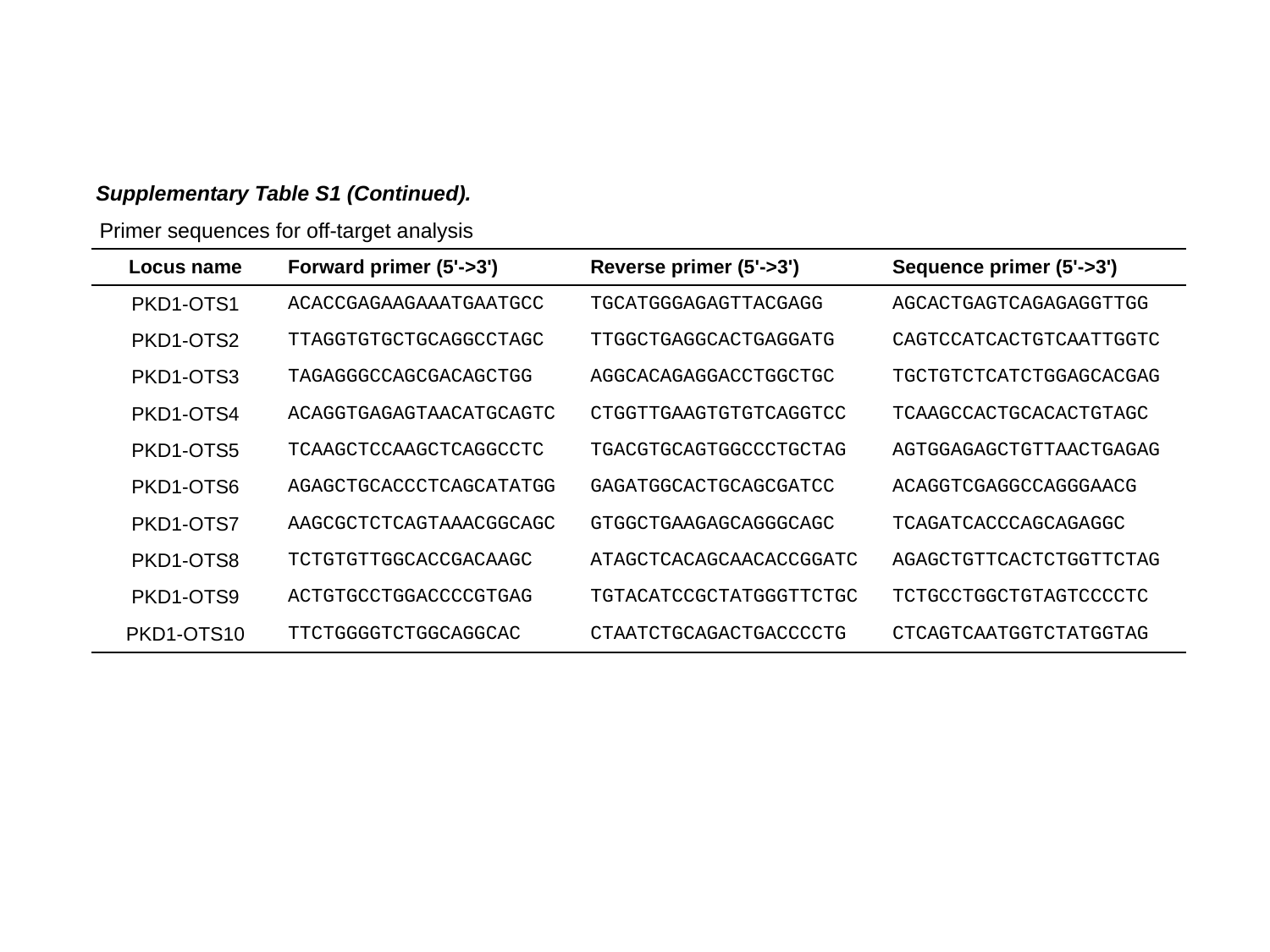

Supplementary Table S1 (Continued).
| Primer sequences for off-target analysis | | | |
| --- | --- | --- | --- |
| Locus name | Forward primer (5'->3') | Reverse primer (5'->3') | Sequence primer (5'->3') |
| PKD1-OTS1 | ACACCGAGAAGAAATGAATGCC | TGCATGGGAGAGTTACGAGG | AGCACTGAGTCAGAGAGGTTGG |
| PKD1-OTS2 | TTAGGTGTGCTGCAGGCCTAGC | TTGGCTGAGGCACTGAGGATG | CAGTCCATCACTGTCAATTGGTC |
| PKD1-OTS3 | TAGAGGGCCAGCGACAGCTGG | AGGCACAGAGGACCTGGCTGC | TGCTGTCTCATCTGGAGCACGAG |
| PKD1-OTS4 | ACAGGTGAGAGTAACATGCAGTC | CTGGTTGAAGTGTGTCAGGTCC | TCAAGCCACTGCACACTGTAGC |
| PKD1-OTS5 | TCAAGCTCCAAGCTCAGGCCTC | TGACGTGCAGTGGCCCTGCTAG | AGTGGAGAGCTGTTAACTGAGAG |
| PKD1-OTS6 | AGAGCTGCACCCTCAGCATATGG | GAGATGGCACTGCAGCGATCC | ACAGGTCGAGGCCAGGGAACG |
| PKD1-OTS7 | AAGCGCTCTCAGTAAACGGCAGC | GTGGCTGAAGAGCAGGGCAGC | TCAGATCACCCAGCAGAGGC |
| PKD1-OTS8 | TCTGTGTTGGCACCGACAAGC | ATAGCTCACAGCAACACCGGATC | AGAGCTGTTCACTCTGGTTCTAG |
| PKD1-OTS9 | ACTGTGCCTGGACCCCGTGAG | TGTACATCCGCTATGGGTTCTGC | TCTGCCTGGCTGTAGTCCCCTC |
| PKD1-OTS10 | TTCTGGGGTCTGGCAGGCAC | CTAATCTGCAGACTGACCCCTG | CTCAGTCAATGGTCTATGGTAG |

## Slide 10
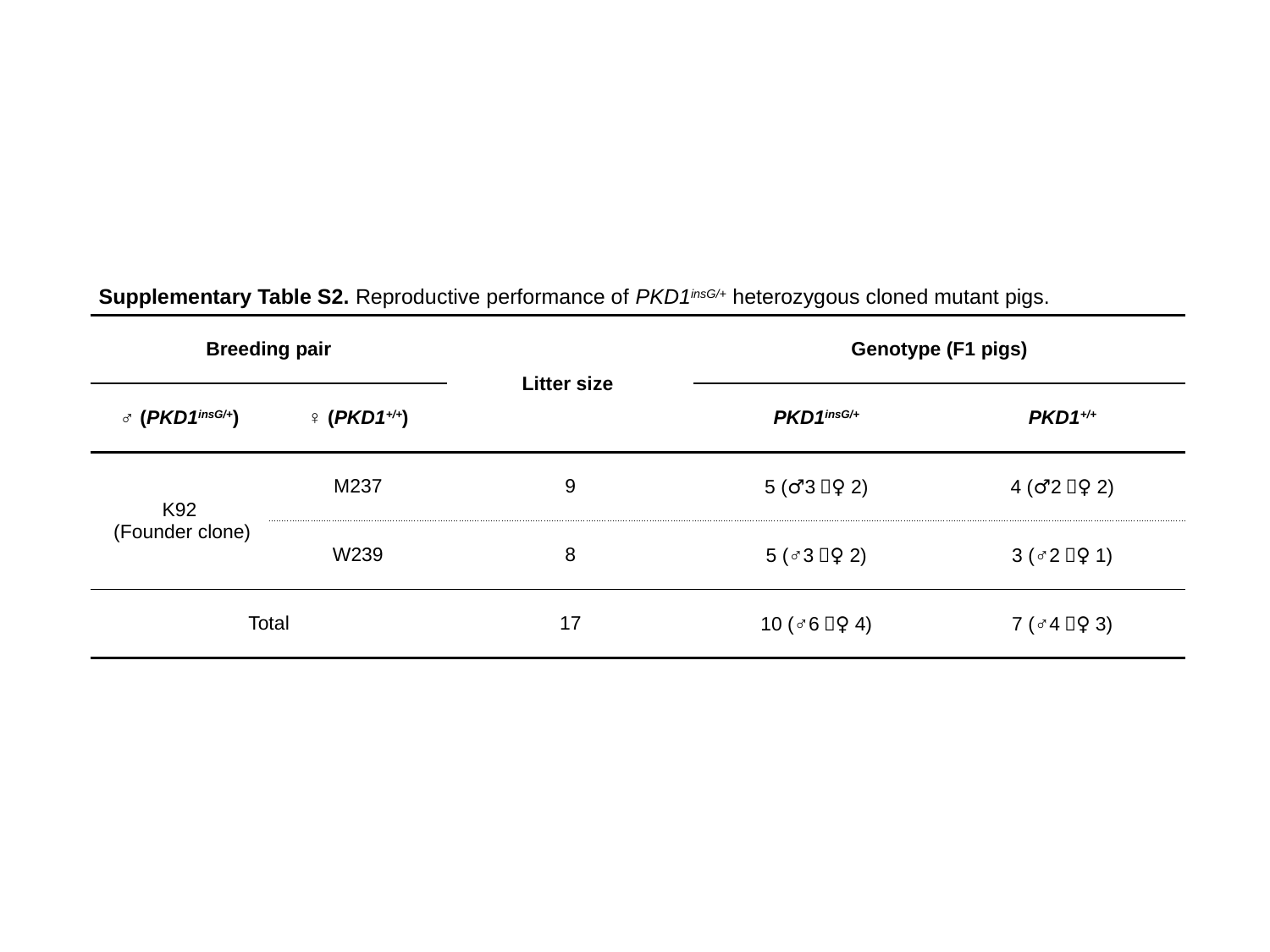

| Supplementary Table S2. Reproductive performance of PKD1insG/+ heterozygous cloned mutant pigs. | | | | |
| --- | --- | --- | --- | --- |
| Breeding pair | | Litter size | Genotype (F1 pigs) | |
| ♂ (PKD1insG/+) | ♀ (PKD1+/+) | | PKD1insG/+ | PKD1+/+ |
| K92 (Founder clone) | M237 | 9 | 5 (♂3：♀2) | 4 (♂2：♀2) |
| | W239 | 8 | 5 (♂3：♀2) | 3 (♂2：♀1) |
| Total | | 17 | 10 (♂6：♀4) | 7 (♂4：♀3) |

## Slide 11
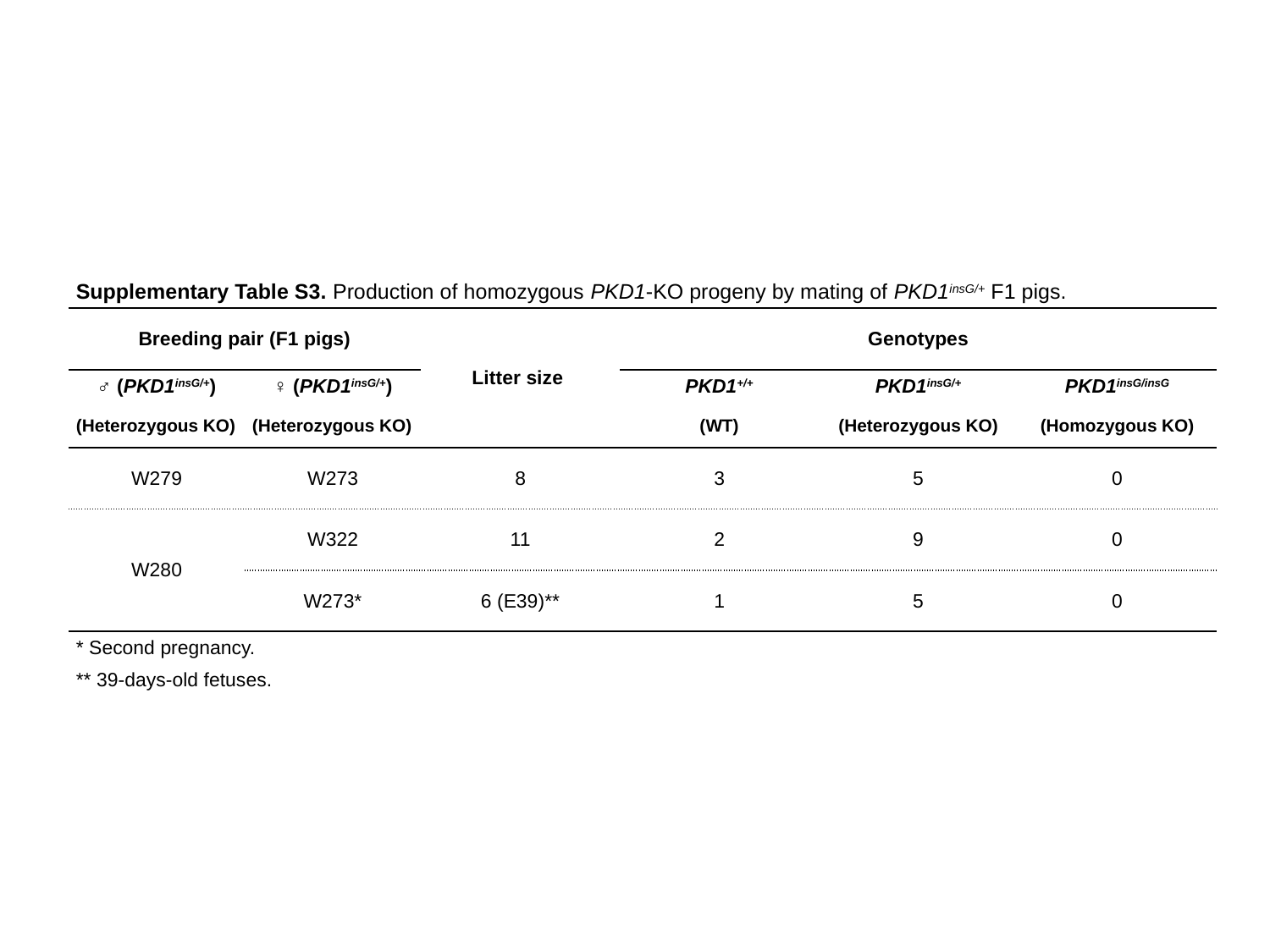

| Supplementary Table S3. Production of homozygous PKD1-KO progeny by mating of PKD1insG/+ F1 pigs. | | | | | |
| --- | --- | --- | --- | --- | --- |
| Breeding pair (F1 pigs) | | Litter size | Genotypes | | |
| ♂ (PKD1insG/+) | ♀ (PKD1insG/+) | | PKD1+/+ | PKD1insG/+ | PKD1insG/insG |
| (Heterozygous KO) | (Heterozygous KO) | | (WT) | (Heterozygous KO) | (Homozygous KO) |
| W279 | W273 | 8 | 3 | 5 | 0 |
| W280 | W322 | 11 | 2 | 9 | 0 |
| | W273\* | 6 (E39)\*\* | 1 | 5 | 0 |
| \* Second pregnancy. | | | | | |
| \*\* 39-days-old fetuses. | | | | | |

## Slide 12
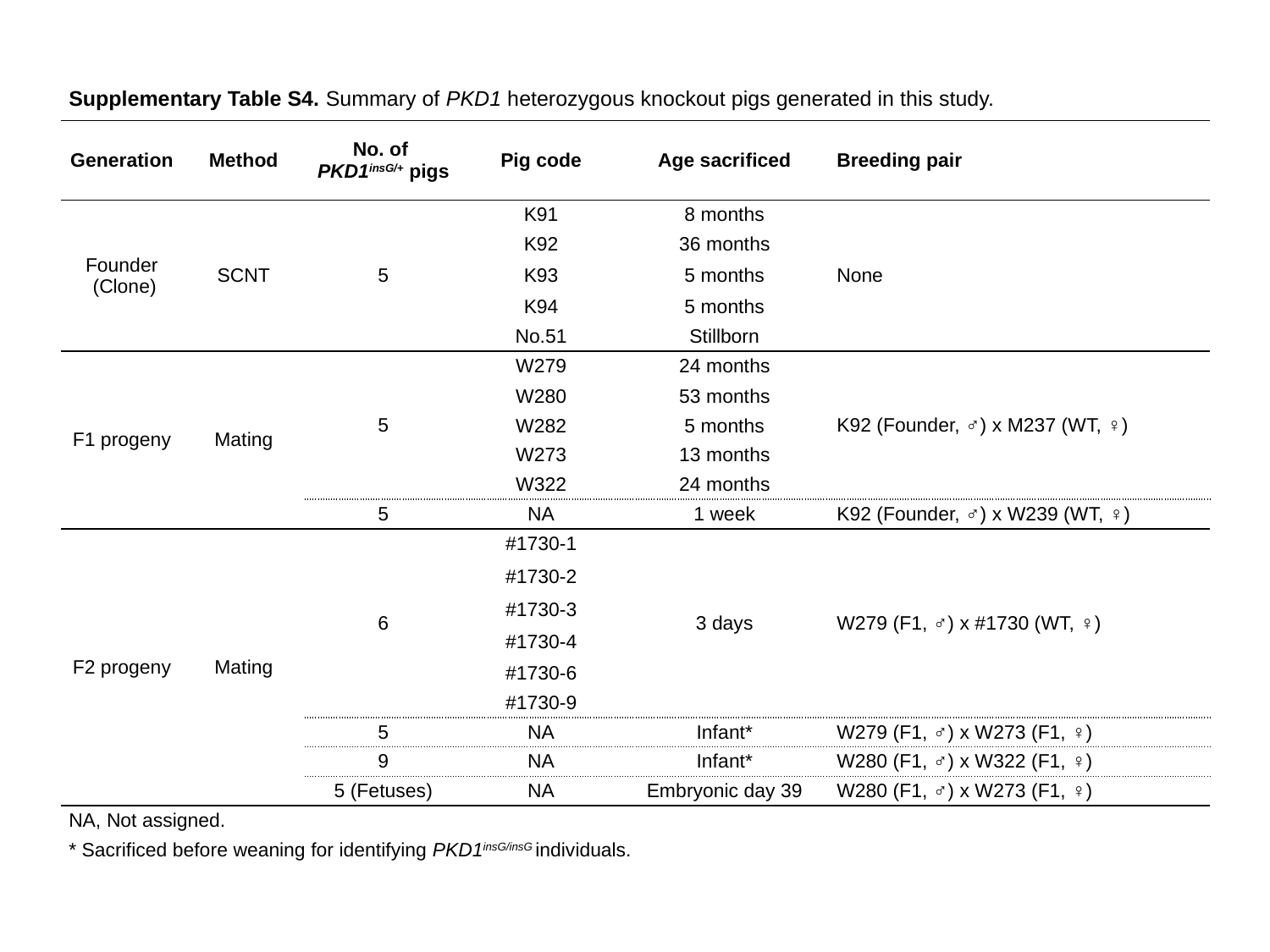

| Supplementary Table S4. Summary of PKD1 heterozygous knockout pigs generated in this study. | | | | | |
| --- | --- | --- | --- | --- | --- |
| Generation | Method | No. of PKD1insG/+ pigs | Pig code | Age sacrificed | Breeding pair |
| Founder (Clone) | SCNT | 5 | K91 | 8 months | None |
| | | | K92 | 36 months | |
| | | | K93 | 5 months | |
| | | | K94 | 5 months | |
| | | | No.51 | Stillborn | |
| F1 progeny | Mating | 5 | W279 | 24 months | K92 (Founder, ♂) x M237 (WT, ♀) |
| | | | W280 | 53 months | |
| | | | W282 | 5 months | |
| | | | W273 | 13 months | |
| | | | W322 | 24 months | |
| | | 5 | NA | 1 week | K92 (Founder, ♂) x W239 (WT, ♀) |
| F2 progeny | Mating | 6 | #1730-1 | 3 days | W279 (F1, ♂) x #1730 (WT, ♀) |
| | | | #1730-2 | | |
| | | | #1730-3 | | |
| | | | #1730-4 | | |
| | | | #1730-6 | | |
| | | | #1730-9 | | |
| | | 5 | NA | Infant\* | W279 (F1, ♂) x W273 (F1, ♀) |
| | | 9 | NA | Infant\* | W280 (F1, ♂) x W322 (F1, ♀) |
| | | 5 (Fetuses) | NA | Embryonic day 39 | W280 (F1, ♂) x W273 (F1, ♀) |
| NA, Not assigned. | | | | | |
| \* Sacrificed before weaning for identifying PKD1insG/insG individuals. | | | | | |
